# Immune and inflammatory features associated with pyroptosis- and myocardial fibrosis-related genes in HFpEF: a machine learning study

**DOI:** 10.3389/fimmu.2026.1925067

**Published:** 2026-10-02

**Authors:** Bo Gao, Gang Wang, Zhi-qiang Xu, Ming Peng

**Affiliations:** 1 Department of Cardiology, Wuhan Fourth Hospital, Wuhan, China; 2 Department of Ultrasound, Wuhan Fourth Hospital, Wuhan, China; 3 Department of Neurosurgery, Wuhan Fourth Hospital, Wuhan, China

**Keywords:** HFPEF, inflammation, machine learning, myocardial fibrosis, *Ptpn11*, pyroptosis

## Abstract

Heart failure with preserved ejection fraction (HFpEF) is a heterogeneous syndrome marked by inflammation, immune dysregulation, and myocardial fibrosis. Whether pyroptosis intersects with fibrotic remodeling in HFpEF remains uncertain. Two murine cardiac transcriptomic datasets were integrated after batch correction and intersected with curated pyroptosis- and myocardial fibrosis-related gene sets. Functional enrichment, univariate logistic regression, support vector machine–recursive feature elimination (SVM-RFE), and least absolute shrinkage and selection operator (LASSO) regression were used for feature selection. Complete separation in the ordinary three-gene logistic fit prompted refitting with Firth bias-reduced logistic regression. Performance was assessed by apparent area under the curve (AUC), stratified bootstrap optimism correction, and repeated nested cross-validation; the panel was re-estimated in a human left-ventricular dataset for exploratory replication. Cell-type Identification by Estimating Relative Subsets of RNA Transcripts (CIBERSORT) estimated immune-cell fractions, and expression was examined in a high-fat diet (HFD)/N^ω^-nitro-L-arginine methyl ester hydrochloride (L-NAME) murine model and whole blood from patients with HFpEF. Six candidates—*Pparg*, *Ace2*, *Smad2*, *Lcn2*, *Ptpn11*, and *Sqstm1*—were identified. Gene Ontology and Kyoto Encyclopedia of Genes and Genomes analyses linked them to SMAD binding, autolysosome/amphisome activity, cellular senescence, efferocytosis, and renin-angiotensin signaling. In the 45-sample discovery cohort, the fixed *Pparg*-*Ptpn11*-*Ace2* Firth model had an apparent AUC of 1.0000; bootstrap correction yielded an AUC of 0.9971 (95% percentile interval, 0.9780–1.0000). Repeated nested cross-validation produced a mean out-of-fold AUC of 0.9286 (2.5%–97.5% percentile interval, 0.8726–0.9700), revealing additional feature-selection uncertainty. Re-estimation in the human dataset represented panel-level replication rather than validation of a locked cross-species model. No inferred immune-cell fraction differed between HFpEF and control after false-discovery-rate correction, although exploratory correlations were observed among deconvolved fractions and with *Ptpn11* expression. HFD/L-NAME-treated mice developed diastolic dysfunction, reduced exercise capacity, pulmonary congestion, cardiomyocyte hypertrophy, and myocardial fibrosis despite preserved systolic function; myocardial *Ptpn11*, *Pparg*, and *Ace2* transcripts and their proteins were reduced. In patients with HFpEF, whole-blood PTPN11 mRNA was lower than in controls and inversely associated with N-terminal pro-B-type natriuretic peptide and C-reactive protein; these unadjusted findings remain exploratory. The three-gene panel is hypothesis-generating rather than a stable diagnostic model; its transportability, selection stability, and clinical relevance require prospective validation.

## 1 Introduction

Heart failure with preserved ejection fraction (HFpEF) is a common, heterogeneous syndrome associated with impaired quality of life, recurrent decompensation, and substantial mortality (1–4). It is diagnosed in patients with heart-failure symptoms or signs despite preserved left ventricular ejection fraction and commonly coexists with hypertension, diabetes, obesity, and other cardiometabolic conditions (3, 5). These comorbidities contribute to myocardial, vascular, and systemic metabolic dysfunction, but the molecular events driving HFpEF remain incompletely defined. Progressive myocardial stiffening and reduced ventricular compliance increase filling pressures and may cause pulmonary congestion, while cardiac reserve can decline before resting systolic function is clearly impaired (6).

Interest in the molecular mechanisms underlying HFpEF has increased substantially in recent years (5). Inflammation has emerged as one possible contributor because it may help explain how systemic comorbidities lead to myocardial remodeling, impaired relaxation, and progressive loss of cardiac function (7). One inflammation-related mechanism that has attracted attention is pyroptosis, a form of programmed cell death characterized by the release of pro-inflammatory signals. Previous studies have linked pyroptosis to myocardial injury, maladaptive cardiac remodeling, and several forms of cardiovascular disease (8, 9). Myocardial fibrosis represents another important feature of HFpEF and can be identified more directly at the histopathological level. Excessive deposition and reorganization of the extracellular matrix increase myocardial stiffness and may worsen cardiac function as the disease progresses (10). Despite their potential biological relationship, pyroptosis and fibrosis have often been investigated independently. It therefore remains unclear whether the two processes are mechanistically connected in HFpEF or simply occur alongside one another. Examining this relationship may improve understanding of disease development and could reveal molecular markers of possible diagnostic value.

Despite advances in HFpEF diagnosis, clinical scores, echocardiography, natriuretic peptides, and invasive hemodynamics have context-dependent limitations and do not directly capture molecular heterogeneity (3, 11). Numerous candidate biomarkers have been proposed, but clinical adoption remains limited by modest accuracy, uncertain reproducibility, and variation across cohorts, laboratories, assay platforms, and analytical strategies (12). Reproducible molecular markers may therefore complement established approaches, provided that their performance is confirmed in independent populations (13).

On the basis of these observations, the present study examined transcriptomic alterations associated with pyroptosis and myocardial fibrosis in HFpEF, with the additional aim of prioritizing molecular features with potential diagnostic relevance. Transcriptomic profiles from independent cohorts were interrogated with machine-learning procedures to isolate discriminative features and fit an exploratory multigene classifier, whose performance was subsequently examined in a separate dataset. To move beyond a purely computational inference, the expression patterns of the selected markers were further assessed in HFpEF mouse models and in clinical specimens obtained from human participants. By juxtaposing bioinformatic screening with animal and human expression assays, the present study sought to sharpen the molecular picture of HFpEF and to nominate candidate biomarkers, together with computationally prioritized molecular leads, for subsequent experimental interrogation.

## 2 Materials and methods

### 2.1 Data acquisition and preprocessing

Two murine cardiac RNA sequencing (RNA-seq) datasets, GSE180065 and GSE194151, were obtained from the Gene Expression Omnibus (GEO) database (https://www.ncbi.nlm.nih.gov/geo/) (14) using the GEOquery package. GSE194151 was generated in the study by Cao et al. (15), whereas GSE180065 is referenced by its GEO accession because no primary publication is currently linked to the GEO record. Both datasets were generated on the Illumina NovaSeq 6000 platform (GPL24247). GSE180065 comprised 15 male cardiac samples: five control samples without HFD/L-NAME exposure (GSM5452204–GSM5452208), five HFpEF samples collected after 5 weeks of HFD/L-NAME treatment (GSM5452209–GSM5452213), and five HFpEF samples collected after 7 weeks of treatment (GSM5452214–GSM5452218). GSE194151 comprised 30 cardiac samples from a 7-week experimental period: the control-diet group included seven females (GSM5829523–GSM5829529) and eight males (GSM5829530–GSM5829537), whereas the HFD/L-NAME group included seven females (GSM5829538–GSM5829544) and eight males (GSM5829545–GSM5829552). No samples belonging to these prespecified control or HFpEF groups were excluded, and all 45 samples were retained for the integrated analysis. Because the HFpEF-to-control ratio differed between GSE180065 (10:5) and GSE194151 (15:15), dataset source and disease status were partially associated in the merged cohort. Differences in induction protocol, treatment duration, and sex composition were therefore treated as residual design heterogeneity when interpreting the integrated analysis. Dataset-level characteristics are summarized in **Table 1**. The original sample-level metadata exported from GEO are provided in **Supplementary Table S1** for GSE194151 and **Supplementary Table S2** for GSE180065. The analytical grouping, treatment duration, sex distribution, and retention of all samples used in the present analysis are described above.

**Table 1:** GEO RNA-seq dataset information.

Items	Dataset 1 (GSE180065)	Dataset 2 (GSE194151)
Platform	GPL24247 (Illumina NovaSeq 6000)	GPL24247 (Illumina NovaSeq 6000)
Species	*Mus musculus*	*Mus musculus*
Tissue	heart tissue	heart tissue
Samples in HFpEF group	10	15
Samples in control group	5	15
Reference	GEO accession: GSE180065	PMID: 35787630

GEO, Gene Expression Omnibus; HfpEF, heart failure with preserved ejection fraction.

Pyroptosis-related genes (PRGs) and myocardial fibrosis-related genes (MFRGs) were primarily assembled from GeneCards (https://www.genecards.org/) (16), which was queried on June 3, 2025 using the terms “Pyroptosis” and “Myocardial Fibrosis,” respectively. No additional relevance-score threshold above zero was imposed: GeneCards records with a Relevance score > 0 were retained, and their Category annotations were recorded. The “Pyroptosis” query returned 835 GeneCards records, of which 534 were annotated as Protein Coding, whereas the “Myocardial Fibrosis” query yielded 636 Protein Coding genes. The PRG catalog was supplemented through a PubMed search performed on June 3, 2025 with the term “Pyroptosis.” Studies were eligible for gene extraction only when they explicitly provided a pyroptosis-related gene list and the reported gene names could be standardized to conventional gene symbols. Reports without an explicit gene list, or whose reported genes could not be matched to standardized gene symbols, were not used for gene extraction. The two eligible published gene lists (17, 18) were extracted, merged with the GeneCards-derived PRGs, standardized, and deduplicated, producing a consolidated catalog of 842 human PRGs before ortholog conversion. Because the discovery transcriptomes were derived from murine myocardium, the human gene sets were converted to mouse orthologs using the human2mouse function in the R package homologene (version 1.4.68.19.3.27), with homologeneData2 used as the mapping database. Human gene symbols were supplied as the input field, and the mapping output retained the corresponding human and mouse gene symbols together with Entrez Gene IDs. For one-to-many relationships, all mapped mouse orthologs were retained; human genes without a mapped mouse ortholog were excluded from the final murine gene sets. Under this mapping procedure, the 842 human PRGs yielded 460 murine PRGs, whereas the 636 human MFRGs yielded 611 murine MFRGs. Their intersection comprised 59 pyroptosis- and myocardial fibrosis-related genes (PMFRGs), which formed the prespecified gene set used for downstream candidate screening (**Supplementary Table S3**; **Supplementary Data S1**, **S2**).

For GSE180065, fragments per kilobase of transcript per million mapped reads (FPKM) expression values supplied in the GEO supplementary files were used, whereas GSE194151 was analyzed from the kallisto-derived FPKM matrix. Ensembl identifiers were converted to gene symbols, and multiple entries mapping to the same gene symbol were collapsed by their mean expression. The resulting matrices were transformed as log_2_(x + 1), restricted to genes shared by the two datasets, and subsequently merged. Dataset source was specified as the batch variable for sva::ComBat adjustment, followed by limma::normalizeBetweenArrays. Both ComBat and subsequent limma analyses were performed on the log_2_-transformed expression matrices rather than on raw counts; a voom workflow was therefore not applied. ComBat was used to attenuate dataset-associated technical variation; it was not assumed to remove the partial association between dataset source and disease status or biological heterogeneity related to the experimental design. Principal component analysis was performed afterward to inspect, in visual terms, whether the batch correction had adequately reduced dataset-specific effects.

### 2.2 Identification of pyroptosis- and myocardial fibrosis-related differentially expressed genes

Samples were classified as either HFpEF or control before differential-expression analysis was performed with the R package limma. For each transcript, log2 fold change (log_2_FC), the raw *P* value, and the Benjamini–Hochberg-adjusted *P* value were retained in the differential-expression output. The primary exploratory screen used |log_2_FC| > 0 and nominal *P* < 0.05. Because this criterion imposes no substantive effect-size restriction and does not control multiplicity, the resulting set was treated as a permissive candidate pool rather than a stringent catalog of DEGs. Threshold dependence was examined separately using two false discovery rate (FDR)-controlled sensitivity criteria: Benjamini–Hochberg-adjusted *P* < 0.05 combined with |log_2_FC| > 0.5, and adjusted *P* < 0.05 without an additional effect-size cutoff. Among them, transcripts with log_2_FC > 0 were labeled as upregulated, whereas those with log_2_FC < 0 were labeled as downregulated. The overall DEG distribution was displayed in a volcano plot constructed with ggplot2.

Candidate pyroptosis- and myocardial fibrosis-related differentially expressed genes (PMFRDEGs) were obtained by intersecting the permissively screened GEO-derived candidate-gene pool with the predefined PMFRG set. Because this intersection arose from a nominal-*P* screening procedure, the resulting genes were carried forward for hypothesis generation rather than regarded as threshold-robust differentially expressed genes. The robustness of this intersection was assessed under the FDR-based sensitivity thresholds described above. Expression differences in PMFRDEGs between HFpEF and control samples were shown in a heatmap generated by pheatmap. Pairwise relationships among PMFRDEGs were then estimated using Spearman’s rank correlation analysis, with the resulting correlation matrix plotted in ggplot2.

### 2.3 GO and KEGG enrichment analyses

Gene Ontology (GO) and Kyoto Encyclopedia of Genes and Genomes (KEGG) over-representation analyses of PMFRDEGs were conducted with the R package clusterProfiler (version 4.10.0). Enrichment significance was evaluated using the hypergeometric test. For GO analysis, enrichGO was applied across the biological process, cellular component, and molecular function ontologies, whereas KEGG analysis was performed using enrichKEGG with the organism argument specified as “mmu”. All genes retained after preprocessing and annotation were used as the background set. Raw *P* values were adjusted for multiple comparisons using the Benjamini–Hochberg procedure. The enrichment output retained pvalue, p.adjust, and qvalue for each term; terms satisfying raw *P* < 0.05 and qvalue < 0.25 were carried forward for exploratory interpretation. The corresponding numerical statistics are reported in **Supplementary Table S4**.

### 2.4 Exploratory feature selection and classifier construction

Feature selection was performed in the merged murine GEO discovery cohort. Each of the six candidate PMFRDEGs was first examined separately using a univariate logistic-regression model fitted with glm using family = binomial(link = “logit”), with control and HFpEF status coded as 0 and 1, respectively. Regression coefficients (β) and Wald 95% confidence intervals were calculated, and genes meeting the two-sided *P* < 0.05 screening criterion were carried forward to support vector machine–recursive feature elimination (SVM-RFE). Because several corresponding odds-ratio estimates were extremely large, **Figure 1A** presents *β* coefficients rather than odds ratios.

**Figure 1:**
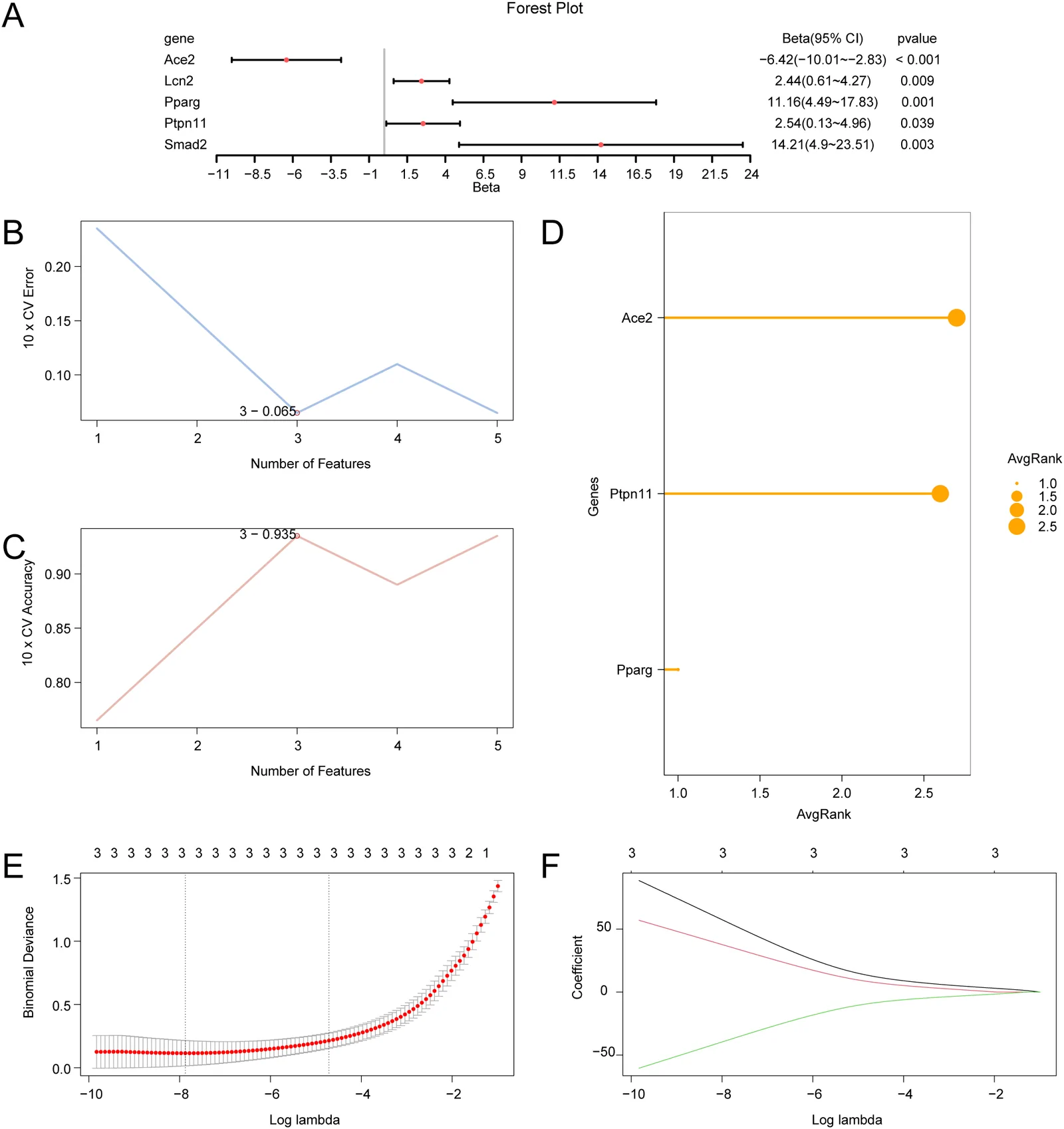
Exploratory feature-selection framework in the merged murine discovery cohort (control, n = 20; HFpEF, n = 25; total n = 45). **(A)** *β* coefficients with Wald 95% confidence intervals from separate univariate logistic-regression models; *β* is expressed on the log-odds scale. A nominal two-sided *P* < 0.05 was used for screening, without multiplicity adjustment. **(B, C)** Candidate feature-set size evaluated by the SVM-RFE resampling procedure, shown as held-out classification error and classification accuracy. **(D)** Aggregated SVM-RFE feature ranking. **(E, F)** LASSO cross-validation and coefficient-path outputs; lambda.min was used to identify nonzero coefficients. Panels B–F represent resampling or model-selection outputs rather than conventional hypothesis tests. Multiplicity adjustment, physical measurement units, and asterisk notation are not applicable to these panels.

SVM-RFE was implemented using the e1071 package (version 1.7-14) within a 10-fold outer resampling scheme. For each outer split used in the original SVM-RFE procedure, feature ranking was recomputed *de novo* from the corresponding training partition, and predictor scaling was estimated from that partition alone. This standardization was confined to SVM-RFE; the final three-gene model used the preprocessed log2 expression values without Z-score standardization. Multiple SVM-RFE (mSVM-RFE) ranking was then performed using five internal resampling folds to generate multiple feature-weight vectors. Within this ranking step, a linear C-classification SVM (kernel = “linear”, cost = 10) was fitted to each resampled training subset. The resulting weight vectors were normalized by their L_2_ norms, and features were ranked according to the ratio of the mean squared weight to its standard deviation. Thus, these five internal resampling folds were used to stabilize feature ranking rather than to tune SVM hyperparameters.

Candidate feature-set sizes were subsequently evaluated across the 10 outer folds using a separate radial-basis-function SVM. For each candidate subset within an outer training fold, gamma values from 2^-12^ to 2^0^ and cost values from 2^-6^ to 2^6^ were tuned by an internal 10-fold cross-validation grid search. The selected gamma and cost combination was then evaluated on the corresponding outer held-out fold. Classification error was averaged across the 10 outer held-out folds, and the feature-set size yielding the lowest mean held-out error was retained. Thus, the radial-SVM tuning procedure was used for candidate-subset evaluation, whereas the linear SVM described above was used for mSVM-RFE feature ranking.

The SVM-RFE-selected genes were subsequently entered into least absolute shrinkage and selection operator (LASSO) logistic regression using glmnet version 4.1-8, with set.seed (2025), family = “binomial”, alpha = 1, and 10-fold cross-validation implemented using cv.glmnet, with binomial deviance as the cross-validation criterion; lambda.min was used to identify genes with nonzero coefficients. The original 10-fold outer resampling procedure applied only to SVM-RFE ranking, recursive elimination, and candidate-subset evaluation; the preceding univariate screen and subsequent LASSO step therefore remained outside that resampling loop. To examine the optimism introduced by these later feature-selection stages, a separate repeated nested cross-validation sensitivity analysis was performed using stratified five-fold outer cross-validation repeated 50 times, yielding 250 outer test folds. Within every outer training set, univariate screening, SVM-RFE, LASSO, and Firth logistic regression were rerun *de novo*, whereas the corresponding outer test fold remained untouched until prediction. This nested analysis started from the six PMFRDEGs identified in the complete 45-sample discovery cohort and consequently does not remove circularity introduced during the upstream candidate-gene discovery step.

### 2.5 Evaluation of the discovery classifier and exploratory replication of the three-gene panel

The fixed discovery model was specified as HFpEF ~ *Pparg* + *Ptpn11* + *Ace2*, with HFpEF coded as 1 and control status as 0. Model predictors were the preprocessed log2 expression values and were not Z-score standardized. Formal separation detection confirmed complete separation in the ordinary maximum-likelihood logistic model; the fit failed to converge and generated warnings that the algorithm did not converge and that fitted probabilities numerically equal to 0 or 1 had occurred. The ordinary maximum-likelihood coefficients were therefore not retained as the final model estimates. Instead, the same three predictors were refitted using Firth bias-reduced logistic regression. The coefficients are reported to document the fitted discovery-cohort model and were rounded to the displayed precision. Model performance was characterized by the apparent area under the curve (AUC) and Brier score and was subsequently corrected for optimism using 2,000 stratified bootstrap resamples with the three predictors fixed. In each bootstrap iteration, the Firth model was refitted and evaluated in both the bootstrap sample and the original discovery cohort; optimism-corrected estimates and bootstrap percentile intervals were then obtained for AUC, Brier score, calibration intercept, and calibration slope. Because separation could also destabilize ordinary calibration regressions, the calibration intercept and slope were estimated using Firth auxiliary models: the original linear predictor was entered as an offset for the intercept estimate, whereas the calibration slope was obtained by regressing outcome on the original linear predictor. The calibration curve, decision-curve analysis, and nomogram were generated from the Firth-model predictions or coefficients and were interpreted as internal model-development outputs rather than evidence of established clinical utility. Between-group expression patterns of *Pparg*, *Ptpn11*, and *Ace2* were visualized, and individual-gene receiver operating characteristic (ROC) curves were generated using the pROC package. Functional relatedness among the three genes was evaluated by GO semantic-similarity analysis using the GOSemSim package.

For exploratory external replication, the HFpEF_2020 human left-ventricular transcriptomic dataset reported by Hahn et al. (19) was analyzed from an FPKM expression matrix. Human gene identifiers were matched to the murine discovery genes through Ensembl ortholog mapping; when multiple identifiers mapped to the same ortholog, their expression values were averaged. The matrix was transformed as log_2_(FPKM + 1), zero-variance genes were removed, and normalization was performed using limma::normalizeBetweenArrays. *PPARG*, *PTPN11*, and *ACE2* were then retained as the prespecified three-gene panel, and a logistic regression model was re-estimated within HFpEF_2020. The coefficients fitted in the murine discovery cohort were not imposed on the human dataset. Accordingly, the ROC, calibration, decision-curve, and nomogram analyses obtained from HFpEF_2020 were interpreted as in-dataset exploratory replication of the three-gene panel rather than external validation of a locked cross-species score.

### 2.6 Immune infiltration analysis

Immune-cell deconvolution was performed with Cell-type Identification by Estimating Relative Subsets of RNA Transcripts (CIBERSORT, v1.03) using the ImmuCC mouse immune-cell signature matrix described by Chen et al. (20), which comprises 511 signature genes representing 25 murine immune-cell types. The reference matrix is mouse-specific but not myocardium-specific and has not been specifically validated in murine HFpEF myocardium. The normalized integrated GEO expression matrix described above was restricted to genes shared with the ImmuCC signature matrix before CIBERSORT was run with perm = 1000 and quantile normalization (QN) = TRUE; the output was interpreted as relative fractions. All 45 discovery samples were retained, and no sample was excluded according to a CIBERSORT *P* value. The archived output retained the 25 relative-fraction columns but not the original per-sample permutation *P* values, Correlation, or root mean square error (RMSE) values; because the random seed of the original run had not been recorded, these quality control (QC) statistics were not reconstructed *post hoc*. B Cells Memory and DC Actived had estimated fractions of zero in every sample and were excluded from downstream comparisons, leaving 23 nonzero cell types. HFpEF-control comparisons were performed using two-sided Wilcoxon rank-sum tests, with Benjamini–Hochberg FDR correction across the 23 cell types and Cliff’s delta with 95% confidence intervals as the effect-size measure. Associations among inferred cell fractions and between model-gene expression and inferred fractions were assessed using two-sided Spearman rank correlation. For the gene–immune-cell bubble plot, *P* values were displayed in four strata: *P* ≤ 0.001, 0.001 < *P* ≤ 0.01, 0.01 < *P* ≤ 0.05, and *P* > 0.05.

### 2.7 Protein–protein interaction network

For the genes retained in the selected model, a protein–protein interaction (PPI) network was generated through the Search Tool for the Retrieval of Interacting Genes/Proteins (STRING) database (https://cn.string-db.org/) (21). The minimum required interaction score was deliberately set at 0.15, allowing relatively broad interaction information to be captured without excluding weak but potentially informative links. From this network, hub PMFRDEGs were screened according to their denser and more pronounced interaction patterns. To broaden the in silico association context beyond the STRING-derived predictions, GeneMANIA (https://genemania.org/) (22) was queried for additional functional relationships assembled from genomic and annotation-based datasets. The resulting PPI structure, together with the predicted functional links, was then visualized and inspected to support downstream biological interpretation.

### 2.8 Construction of the regulatory network

Transcription factors (TFs) with potential regulatory effects on the hub PMFRDEGs were predicted by querying the ChIPBase Database (http://rna.sysu.edu.cn/chipbase/) (23). On the basis of these predicted TF-mRNA relationships, a regulatory network was assembled and rendered in Cytoscape. Candidate drugs or chemical compounds associated with the hub PMFRDEGs were then retrieved from the Comparative Toxicogenomics Database (CTD) (https://ctdbase.org/) (24), where curated chemical–gene associations provide a useful reference for downstream inference. Using these CTD-derived records, a drug-gene interaction network was constructed in Cytoscape and visually examined.

### 2.9 Molecular docking analyses

#### 2.9.1 Protein–protein docking of PPARG and PTPN11

Protein–protein docking was performed locally under Linux using HDOCKlite v1.1 between full-length mouse PPARG and a PTPN11 structural construct comprising residues 4–103. The PTPN11 structural input was derived from Protein Data Bank (PDB) entry 1AYA (UniProt P35235), with residues 4–103 retained for docking, whereas PPARG was represented by the full-length AlphaFold model AF-P37238-F1 corresponding to UniProt P37238 (residues 1–505). One hundred candidate docking poses were generated, and model_1, the top-ranked pose according to the HDOCK scoring function, was retained for subsequent structural analysis. The HDOCK docking score and confidence score were treated as model-ranking and docking-confidence metrics rather than thermodynamic binding free energies.

The retained top-ranked docking model (model_1) was further evaluated by molecular mechanics/generalized Born surface area (MM/GBSA) analysis using HawkDock with its default MM/GBSA settings. The calculation employed the ff02 force field together with the GB^OBC1^ implicit-solvent model and an interior dielectric constant of 1. Before energy evaluation, the retained modeled protein–protein complex was minimized for 5,000 steps, consisting of 2,000 cycles of steepest-descent minimization followed by 3,000 cycles of conjugate-gradient minimization; a cutoff distance of 999 Å was used for van der Waals interactions. The MM/GBSA estimate comprised van der Waals, electrostatic, polar-solvation, and nonpolar-solvation contributions. No separate configurational-entropy term was estimated. The resulting ΔG_MM/GBSA_ value was treated as a model-dependent energetic estimate and was interpreted separately from the HDOCK docking score and confidence score.

The retained docking pose was subsequently analyzed using the Protein–Ligand Interaction Profiler (PLIP) web server to annotate putative residue-level contacts at the modeled interface. In the analyzed docking complex, full-length PPARG was assigned as chain A, whereas the PTPN11 structural construct comprising residues 4–103 was assigned as chain B. PyMOL version 2.5 was used for structural visualization.

#### 2.9.2 Protein–ligand docking of PPARG-associated compounds

For the separate protein–ligand docking analysis, the two-dimensional structures of the candidate compounds associated with PPARG were retrieved from PubChem (http://pubchem.ncbi.nlm.nih.gov/) (25), and the corresponding PubChem compound identifiers were recorded for traceability. These ligand structures were then transformed into energy-minimized three-dimensional conformations in ChemOffice and exported in.mol2 format. For the protein targets, experimentally resolved crystal structures were downloaded from the Research Collaboratory for Structural Bioinformatics Protein Data Bank (http://www.rcsb.org/) (26). Before receptor preparation, the Protein Data Bank accession number, selected chain, and crystallographic resolution were recorded for each structure. Receptor files were then prepared with PyMOL version 2.5. In this procedure, crystallographic water molecules were deleted, incomplete side chains were repaired when needed, and cofactors or metal ions were kept when they were likely to contribute to ligand recognition.

Ligand and receptor structures were first processed in AutoDock Tools and subsequently exported in the Protein Data Bank format containing partial atomic charges and AutoDock atom types (PDBQT). During this preparation step, hydrogen atoms and partial atomic charges were assigned, and the rotatable bonds of each ligand were specified. For the receptors, docking grids were positioned over active sites that had either been experimentally characterized or computationally inferred, rather than being placed on arbitrary protein regions. Protein–ligand docking calculations were performed with AutoDock Vina. For each protein–ligand pair, the docking search space was defined to encompass the predefined PPARG binding pocket. Among the resulting poses, the pose with the lowest AutoDock Vina score located within that pocket was retained for subsequent interaction analysis.

The highest-ranked protein–ligand complexes were examined using Discovery Studio 2019 and PyMOL version 2.5. Two-dimensional interaction diagrams and three-dimensional structural models were generated to visualize hydrogen bonding, hydrophobic contacts, and other noncovalent interactions involving key amino acid residues. AutoDock Vina scores were interpreted as relative docking scores for exploratory ranking rather than as experimentally determined binding free energies.

### 2.10 Molecular dynamics simulations

#### 2.10.1 Molecular dynamics simulations of the modeled PPARG–PTPN11 complex

The retained HDOCK model_1 complex comprising full-length PPARG (chain A) and the PTPN11 structural construct comprising residues 4–103 (chain B) was subjected to three independent 100-ns molecular dynamics simulations using GROningen MAchine for Chemical Simulations (GROMACS) version 2022.2. Both protein chains were parameterized using the Amber99SB-ILDN force field. All three replicate trajectories were initiated from the same HDOCK model_1 structure, while different random seeds were used to generate different initial velocities for the three simulations. Each system was solvated using the Transferable Intermolecular Potential with 3 Points (TIP3P) water model and adjusted to 0.15 M NaCl while maintaining overall charge neutrality. Long-range electrostatic interactions were treated using the particle mesh Ewald (PME) method, with a 1.2-nm cutoff applied to van der Waals and short-range Coulomb interactions. Bond lengths were constrained using the LINear Constraint Solver (LINCS), and a 2-fs integration time step was used. Each replicate underwent 1 ns of constant number of particles, volume, and temperature (NVT) equilibration followed by 1 ns of constant number of particles, pressure, and temperature (NPT) equilibration at 300 K and 1 bar before the 100-ns production simulation.

For trajectory analysis, periodic-boundary artifacts were first corrected, and the whole-complex backbone was least-squares fitted to the starting HDOCK model_1 reference structure before calculation of overall backbone root mean square deviation (RMSD). For interface-specific analysis, interface residues were defined from the starting HDOCK model_1 structure as residues containing at least one heavy atom within 4.0 Å of a heavy atom in the opposing chain. The resulting interface residue set was kept fixed for all subsequent interface RMSD analyses. Before interface RMSD calculation, periodic-boundary artifacts were corrected and the trajectories were subjected to least-squares fitting/alignment against the starting reference complex. Center-of-mass separation was calculated between all atoms of PPARG chain A and all atoms of PTPN11 chain B. Intermolecular hydrogen bonds between the two modeled chains were quantified using a donor–acceptor distance cutoff of 0.35 nm and an angular cutoff of 30°. Radius of gyration (R**_g_**), solvent-accessible surface area (SASA), per-residue root mean square fluctuation (RMSF), and projected free-energy surfaces were additionally evaluated to characterize global and local trajectory behavior.

#### 2.10.2 Molecular dynamics simulation of the PPARG–troglitazone complex

Molecular dynamics simulation of the PPARG–troglitazone protein–ligand complex was carried out with GROMACS 2022.2, using the Amber99SB-ILDN force field for PPARG (topology generated via pdb2gmx) and General AMBER Force Field 2 (GAFF2) parameters for troglitazone, built with sobtop_1.0 (dev3.1); ligand atomic charges were assigned using the restrained electrostatic potential (RESP) scheme. The complex was solvated in a cubic Transferable Intermolecular Potential with 3 Points (TIP3P) water box extending at least 1.2 nm from the protein surface, and Na^+^/Cl^-^ ions were introduced with gmx genion to reach a 0.15 M NaCl environment while neutralizing net charge (27). Long-range electrostatics were handled by the particle mesh Ewald (PME) method, with van der Waals and short-range Coulombic interactions truncated at 1.2 nm; bond lengths were constrained via LINCS, and a 2-fs integration time step was adopted throughout. Before the production run, the system underwent energy minimization by steepest-descent followed by conjugate-gradient minimization. This was followed by 1 ns of NVT equilibration and 1 ns of NPT equilibration with position restraints on both the protein and ligand; temperature and pressure were coupled using the V-rescale thermostat and Parrinello–Rahman barostat, respectively. All restraints were then removed for the 100-ns production simulation. Post-simulation analysis used the built-in GROMACS tools. Backbone RMSD, protein–ligand separation, per-residue RMSF, R_g_, SASA, and intermolecular hydrogen bonds were evaluated using gmx rms, gmx distance, gmx rmsf, gmx gyrate, gmx sasa, and gmx hbond, respectively. A two-dimensional projected free-energy surface based on backbone RMSD and R_g_ was generated using gmx sham to characterize the sampled conformational distribution.

### 2.11 Construction of HFpEF animal models

Wuhan Luobin Life Technology Co., Ltd. is a privately owned enterprise and holds an Experimental Animal Use License [SYXK (Hubei) 2024-0149]. All animal procedures were reviewed and approved by the Ethics Committee of Wuhan Luobin Life Technology Co., Ltd. (approval no. LBSM2025083), which is responsible for the ethical review of animal-experiment projects. All animal husbandry, HFpEF model establishment, functional testing, and terminal tissue collection were carried out at Wuhan Luobin Life Technology Co., Ltd. No separate animal-ethics approval was obtained from Wuhan Fourth Hospital or from any other author-affiliated or collaborating institution. The experiments were performed in accordance with the Guide for the Care and Use of Laboratory Animals. Twelve eight-week-old male C57BL/6J mice were included, with six animals allocated to each experimental group. No formal *a priori* power calculation was performed; the group size was determined before experimentation on the basis of prior experience with the HFD/L-NAME HFpEF model, the anticipated reproducibility of its physiological and histopathological phenotype, and the principle of limiting animal use while retaining adequate biological replication for exploratory comparisons. Following acclimatization, mice were assigned in a 1:1 ratio to the standard-chow control group or the HFD/L-NAME group according to a computer-generated random sequence. Because the dietary and L-NAME interventions were readily distinguishable, personnel responsible for routine treatment administration were not blinded to group allocation. Investigators performing quantitative echocardiographic and histological assessments, however, remained blinded to group identity until completion of the corresponding measurements. The HFD/L-NAME intervention consisted of a high-fat diet (HFD) providing 60% kcal from fat, in combination with N^ω^-nitro-L-arginine methyl ester hydrochloride (L-NAME, 0.5 g/L) administered in the drinking water for 15 weeks (28). Body weight was measured once weekly. Exercise tolerance was examined by an exhaustion test (29). Cardiac function was assessed by transthoracic echocardiography using a Vevo 3100 imaging platform equipped with an MS400 probe (VisualSonics Inc.) (28). For each echocardiographic parameter, measurements from three consecutive technically acceptable cardiac cycles were averaged to generate a single value for each animal. Offline quantification was performed using Vevo LAB 5.7.1 software by an investigator blinded to treatment allocation. At the completion of the experimental period, cardiac tissues and serum were harvested and preserved at −80 °C until subsequent biochemical assays and histopathological evaluation were undertaken.

### 2.12 Treadmill exhaustion test

Before the formal assessment, mice were familiarized with treadmill running for three consecutive days on a treadmill system (ZH-PT/5S, Zhenghua Biotechnology, China). During this adaptation period, animals ran at a 0° incline under a low-intensity workload, so that the subsequent test would be less influenced by novelty-related stress. At week 12 of the intervention, a treadmill exhaustion test was conducted with the incline set to 20°. The running speed was initially fixed at 5 m/min; after 4 min, it was raised to 14 m/min, and thereafter increased by 2 m/min at 2-min intervals until exhaustion occurred. Electrical stimulation was set at 0.8 mA and applied solely as a prompt to maintain running behavior. Exhaustion was defined by either of the following criteria: failure to leave the stimulation zone after 20 stimuli delivered within 10 s, or an evident inability to sustain coordinated running despite stimulation. Once either condition was observed, the trial was stopped at once. The total running distance was recorded and used as the measure of exercise capacity.

### 2.13 Patient recruitment and sample collection

The study protocol was approved by the Ethics Committee of Wuhan Fourth Hospital (approval no. KY2025-128-02). All participants provided written informed consent before sample collection. From December 2025 to February 2026, 37 inpatients were recruited from the Department of Cardiology, including 19 patients with HFpEF and 18 contemporaneous control participants. Control participants were selected by convenience sampling from cardiology inpatients during the same recruitment period. The HFpEF and control groups were not matched for age, sex, body mass index, renal function, comorbidities, or medication use. Baseline demographic and clinical characteristics are summarized in **Table 2**.

**Table 2:** Clinical characteristics of HfpEF and control groups.

Characteristic	Control (n = 18)	HFpEF (n = 19)	*P* value
Age, years	64.78 ± 10.06	79.37 ± 6.15	< 0.0001 ^b^
Female sex, n (%)	11 (61.11%)	11 (57.89%)	> 0.9999 ^d^
Body mass index, kg/m²	24.14 ± 2.20	22.66 ± 3.54	0.1393 ^a^
Systolic blood pressure, mmHg	132.00 (124.25, 143.25)	135.00 (120.00, 145.00)	0.9101 ^c^
Diastolic blood pressure, mmHg	81.78 ± 10.20	74.63 ± 9.51	0.0341 ^a^
Heart rate, bpm	75.39 ± 8.62	76.42 ± 14.57	0.7936 ^b^
NYHA III, n (%)	0 (0.00%)	11 (57.89%)	0.0001 ^d^
Past medical history			
Hypertension, n (%)	11 (61.11%)	16 (84.21%)	0.1510 ^d^
Diabetes, n (%)	6 (33.33%)	11 (57.89%)	0.1914 ^d^
Coronary artery disease, n (%)	6 (33.33%)	13 (68.42%)	0.0502 ^d^
Atrial fibrillation or flutter, n (%)	0 (0.00%)	3 (15.79%)	0.2297 ^d^
Stroke, n (%)	1 (5.56%)	7 (36.84%)	0.0422 ^d^
Hyperlipidemia, n (%)	2 (11.11%)	3 (15.79%)	> 0.9999 ^d^
Medications			
ACEI, ARB, or ARNI, n (%)	8 (44.44%)	8 (42.11%)	> 0.9999 ^d^
Beta-blocker, n (%)	3 (16.67%)	8 (42.11%)	0.1510 ^d^
Diuretic, n (%)	1 (5.56%)	9 (47.37%)	0.0078 ^d^
Aldosterone antagonist, n (%)	0 (0.00%)	8 (42.11%)	0.0031 ^d^
SGLT2 inhibitor, n (%)	1 (5.56%)	4 (21.05%)	0.3398 ^d^
Statin, n (%)	7 (38.89%)	13 (68.42%)	0.1031 ^d^
Laboratory data			
NT-proBNP, pg/mL	47.81 (26.69, 56.64)	2077.78 (634.17, 3781.73)	< 0.0001 ^c^
Hemoglobin, g/L	133.06 ± 9.25	118.05 ± 18.64	0.0043 ^b^
WBC (#), ×10^9^/L	6.11 (4.52, 7.06)	7.27 (5.46, 8.93)	0.2809 ^c^
NEU (#), ×10^9^/L	3.95 (2.84, 5.41)	5.83 (3.53, 7.21)	0.1760 ^c^
LYM (#), ×10^9^/L	1.26 (1.12, 1.55)	1.05 (0.73, 1.30)	0.0874 ^c^
MON (#), ×10^9^/L	0.38 ± 0.13	0.48 ± 0.24	0.1212 ^b^
EOS (#), ×10^9^/L	0.10 (0.05, 0.14)	0.07 (0.02, 0.18)	0.6902 ^c^
BAS (#), ×10^9^/L	0.02 (0.02, 0.03)	0.02 (0.01, 0.03)	0.1956 ^c^
Creatinine, µmol/L	63.60 (54.63, 74.38)	106.70 (72.80, 134.40)	0.0011 ^c^
eGFR, mL/min/1.73 m²	92.56 ± 14.23	55.16 ± 22.85	< 0.0001 ^a^
Alanine aminotransferase, U/L	21.50 (15.00, 32.75)	14.00 (11.00, 28.00)	0.1125 ^c^
Aspartate aminotransferase, U/L	20.00 (17.00, 33.50)	20.00 (18.00, 38.00)	0.7575 ^c^
Hemoglobin A1c, %	5.85 (5.78, 6.60)	6.10 (5.63, 6.45)	0.8454 ^c^
Total cholesterol, mmol/L	4.47 ± 1.36	3.81 ± 1.27	0.1442 ^a^
Low-density lipoprotein cholesterol, mmol/L	2.39 (1.74, 2.82)	1.69 (1.28, 2.43)	0.0744 ^c^
Triglycerides, mmol/L	1.36 (1.16, 1.88)	0.82 (0.52, 1.42)	0.0207 ^c^
Fasting blood glucose, mmol/L	6.77 (5.52, 8.06)	5.86 (4.84, 9.70)	0.4147 ^c^
C-reactive protein, mg/L	2.48 (0.79, 3.94)	17.20 (1.64, 78.74)	0.0067 ^c^
Echocardiography data			
LVEF, %	64.22 ± 4.73	59.79 ± 5.68	0.0146 ^a^
LVFS, %	35.00 (30.75, 36.00)	31.00 (29.75, 35.25)	0.0652 ^c^
Left atrial internal diameter, cm	3.19 ± 0.50	3.64 ± 0.64	0.0232 ^a^
Left ventricular internal diameter, cm	4.34 ± 0.42	4.49 ± 0.49	0.3315 ^a^
Right atrial internal diameter, cm	3.40 (3.30, 3.60)	3.55 (3.38, 3.80)	0.0885 ^c^
Right ventricular internal diameter, cm	3.17 ± 0.36	3.29 ± 0.23	0.2387 ^a^
Interventricular septal thickness, cm	1.00 (0.98, 1.23)	1.10 (0.90, 1.30)	0.3208 ^c^
E/A ratio	0.80 (0.60, 1.10)	0.80 (0.60, 1.40)	0.5356 ^c^
E/e′ ratio	8.75 (7.10, 11.55)	11.30 (7.67, 13.80)	0.2542 ^c^

Data are presented as n (%), mean ± standard deviation, or median (25th–75th percentile), as appropriate. Superscript symbols identify the statistical test used for each comparison: ^a^two-sided Student’s t-test; ^b^two-sided Welch’s t-test; ^c^two-sided Mann–Whitney U test; and ^d^two-sided Fisher’s exact test. No adjustment for multiple comparisons was applied to the descriptive between-group comparisons in **Table 2**; all reported *P* values are nominal and two-sided. The overall cohort comprised 18 controls and 19 patients with HFpEF; variable-specific sample sizes were smaller when measurements were unavailable. For HFpEF cohort eligibility, an NT-proBNP concentration ≥ 125 pg/mL was used as the objective biomarker threshold. Echocardiographic structural variables were recorded for phenotypic characterization and were not used as separate mandatory inclusion thresholds. An E/e′ ratio ≥ 9 was used to define echocardiographic evidence of diastolic dysfunction. ACEI, angiotensin-converting enzyme inhibitor; ARB, angiotensin II receptor blocker; ARNI, angiotensin receptor-neprilysin inhibitor; SGLT2, sodium-glucose cotransporter 2; eGFR, estimated glomerular filtration rate; WBC (#), white blood cell count; NEU (#), absolute neutrophil count; LYM (#), absolute lymphocyte count; MON (#), absolute monocyte count; EOS (#), absolute eosinophil count; BAS (#), absolute basophil count. The symbol # denotes an absolute cell count; WBC and the absolute leukocyte subtype counts are expressed in × 10^9^/L. LVEF, left ventricular ejection fraction; LVFS, left ventricular fractional shortening; E/A, early diastolic mitral inflow velocity/atrial contraction mitral inflow velocity; E/e′, early mitral inflow velocity/early diastolic mitral annular velocity; NYHA, New York Heart Association; NT-proBNP, N-terminal pro-B-type natriuretic peptide; HFpEF, heart failure with preserved ejection fraction.

HFpEF cohort eligibility was defined using a study-specific clinical framework informed by established consensus recommendations (30–32). Eligible patients had exertional dyspnea consistent with New York Heart Association (NYHA) functional class II or III, clinical evidence of heart failure, a preserved left ventricular ejection fraction (LVEF ≥ 50%), and an N-terminal pro-B-type natriuretic peptide (NT-proBNP) concentration ≥125 pg/mL. Echocardiographic indices of diastolic function and cardiac structure were used to support phenotypic characterization rather than as independent mandatory enrollment criteria. An early mitral inflow velocity-to-early diastolic mitral annular velocity ratio (E/e′) ≥9 was considered evidence of diastolic dysfunction. Structural echocardiographic variables, including interventricular septal thickness and left atrial internal diameter, were recorded for phenotypic characterization but were not subjected to separate mandatory thresholds for cohort inclusion. Among the 19 patients with HFpEF, all 19 had LVEF ≥ 50% and NT-proBNP ≥ 125 pg/mL, 11 had E/e′ ≥ 9, 8 were in NYHA functional class II, and 11 were in NYHA functional class III. The same exclusion criteria were applied to both HFpEF and control participants. Participants were excluded if they had an LVEF < 50%, congenital heart disease, hypertrophic cardiomyopathy, a history of heart transplantation, constrictive pericarditis, severe valvular disease, or infiltrative or restrictive cardiomyopathy. Control participants had no clinical signs or symptoms of heart failure, an LVEF ≥ 50%, and an NT-proBNP concentration <125 pg/mL.

For both HFpEF and control participants, fasting venous whole blood was collected immediately after admission following an overnight fast. Blood was drawn into tubes containing ethylenediaminetetraacetic acid (EDTA), and whole-blood samples were used directly for RNA extraction without plasma separation or prior isolation of peripheral-blood cells.

### 2.14 RNA extraction and reverse-transcription quantitative polymerase chain reaction

Total RNA was isolated from murine cardiac tissue and human whole blood with TRIzol reagent, after which the extracted RNA was reverse-transcribed into complementary DNA using a Roche reverse-transcription kit. RT-qPCR was then carried out with Roche SYBR Green chemistry; primer sequences for the murine and human assays are provided in **Supplementary Tables S5**, **S6**, respectively. Glyceraldehyde-3-phosphate dehydrogenase (GAPDH) served as the endogenous reference, with *Gapdh* and *GAPDH* used for the murine and human assays, respectively; relative expression of the target transcripts was determined using the 2^−ΔΔ^*^Ct^* method.

### 2.15 Western blotting

Cardiac tissue samples were lysed in lysis buffer, and the extracted proteins were mixed with loading buffer before heat denaturation for 5 min. Equal amounts of protein were separated by 10% sodium dodecyl sulfate–polyacrylamide gel electrophoresis and transferred onto polyvinylidene fluoride membranes. The membranes were incubated at 4 °C overnight with primary antibodies against GAPDH (1:100,000; 60004-1-Ig; Proteintech), PTPN11 (1:2,000; 66795-1-Ig; Proteintech), PPARG (1:10,000; 81490-5-RR; Proteintech), and ACE2 (1:4,000; 66699-1-Ig; Proteintech). After several washes, the membranes were exposed to the corresponding secondary antibodies for 1 h at room temperature. Protein bands were visualized using an enhanced chemiluminescence detection system. Band intensities were quantified by densitometry, and the signal intensity of each target protein was normalized to the GAPDH signal from the corresponding lane. The resulting GAPDH-adjusted ratio for each mouse was used directly as the animal-level measurement for statistical analysis, without further rescaling to the control-group mean.

### 2.16 Heart histology

Excised hearts were fixed in 10% neutral buffered formalin, processed through standard paraffin-embedding procedures, and cut into 5 μm sections for subsequent histopathological examination. After deparaffinization, the sections were stained with hematoxylin and eosin (H&E; C0105S, Beyotime Biotechnology) and Masson’s trichrome (C0189M, Beyotime Biotechnology) kits, in accordance with the manufacturers’ instructions. H&E staining was used to examine the overall myocardial architecture and cellular morphology, whereas Masson’s trichrome staining was performed to visualize collagen accumulation and estimate the degree of myocardial fibrosis. For quantitative histomorphometry, three nonadjacent cardiac sections were analyzed from each animal. Three nonoverlapping microscopic fields were sampled from each section using the same predefined field-selection procedure across animals. H&E-stained sections were used to quantify cardiomyocyte cross-sectional area, whereas Masson’s trichrome-stained sections were used to determine myocardial fibrotic area. Images were quantified using ImageJ software (National Institutes of Health, USA) by an investigator blinded to experimental allocation. Measurements from individual fields were first averaged within each section and subsequently across sections, so that each animal contributed a single biological value to the statistical analysis.

### 2.17 Immunohistochemistry

Paraffin-embedded sections were deparaffinized, passed through graded alcohols for rehydration, and then subjected to antigen retrieval. Endogenous peroxidase activity was quenched for 15 min. To reduce nonspecific antibody binding, the sections were blocked with 5% bovine serum albumin for 1 h at room temperature. The blocked samples were subsequently incubated overnight with primary antibodies against PTPN11 (1:300; 66795-1-Ig; Proteintech), PPARG (1:2,000; 81490-5-RR; Proteintech), and ACE2 (1:2,000; 66699-1-Ig; Proteintech). After treatment with the corresponding secondary antibodies for 1 h at 37 °C, immunoreactive signals were visualized using a 3,3′-diaminobenzidine (DAB) detection kit, and nuclei were counterstained with hematoxylin. For quantitative immunohistochemical analysis, three nonadjacent sections were examined from each animal, with three nonoverlapping microscopic fields randomly selected from each section for quantitative analysis. Images were acquired under identical microscopic conditions and analyzed with ImageJ software (National Institutes of Health, USA) by an investigator blinded to experimental allocation. Before quantification, a stain-free region of each image was sampled to establish the incident/background reference for optical-density calibration. DAB-positive regions were then delineated by color-based segmentation, and integrated optical density (IOD) was recorded for each field. The calibrated IOD values were analyzed on their native scale without further normalization to positive-staining area or to the control-group mean. Field-level IOD measurements were first averaged within each section and subsequently across the three sections, yielding one animal-level value for each protein. Individual fields and sections were not treated as independent biological replicates.

### 2.18 Statistical analysis

Statistical analyses were performed using R software (version 4.2.2) or GraphPad Prism 10.6.0. For the baseline comparisons presented in **Table 2**, continuous variables were summarized and compared according to their distributional characteristics. Normality was assessed separately within the control and HFpEF groups using the Shapiro–Wilk test. A variable was treated as approximately normally distributed when the Shapiro–Wilk test did not indicate a departure from normality in either group (*P* ≥ 0.05 in both groups). Such variables were reported as mean ± standard deviation. Equality of variances was assessed using a two-sided *F* test. Student’s t-test was used for two-group comparisons when variances were equal (*P* ≥ 0.05), whereas Welch’s t-test was used when variances were unequal (*P* < 0.05). Variables for which the Shapiro–Wilk test indicated a departure from normality in at least one group (*P* < 0.05) were summarized as median (25th–75th percentile) and compared using a two-sided Mann–Whitney U test. Categorical variables were presented as counts and percentages and were compared using two-sided Fisher’s exact tests. The same distribution- and variance-based decision rule was applied to single-time-point animal-level comparisons in **Figure 2**. Approximately normally distributed data were compared using a two-sided Student’s t-test when variances were equal and a two-sided Welch’s t-test when variances were unequal; non-normally distributed data were analyzed using a two-sided Mann–Whitney U test. The animal was treated as the biological unit. Longitudinal body-weight measurements in **Figure 2A** were analyzed using two-way repeated-measures analysis of variance (ANOVA) with time and group as factors and animal as the repeated factor. The Geisser–Greenhouse correction was applied without assuming sphericity, and between-group comparisons at individual time points were adjusted using the Šídák multiple-comparisons procedure.

**Figure 2:**
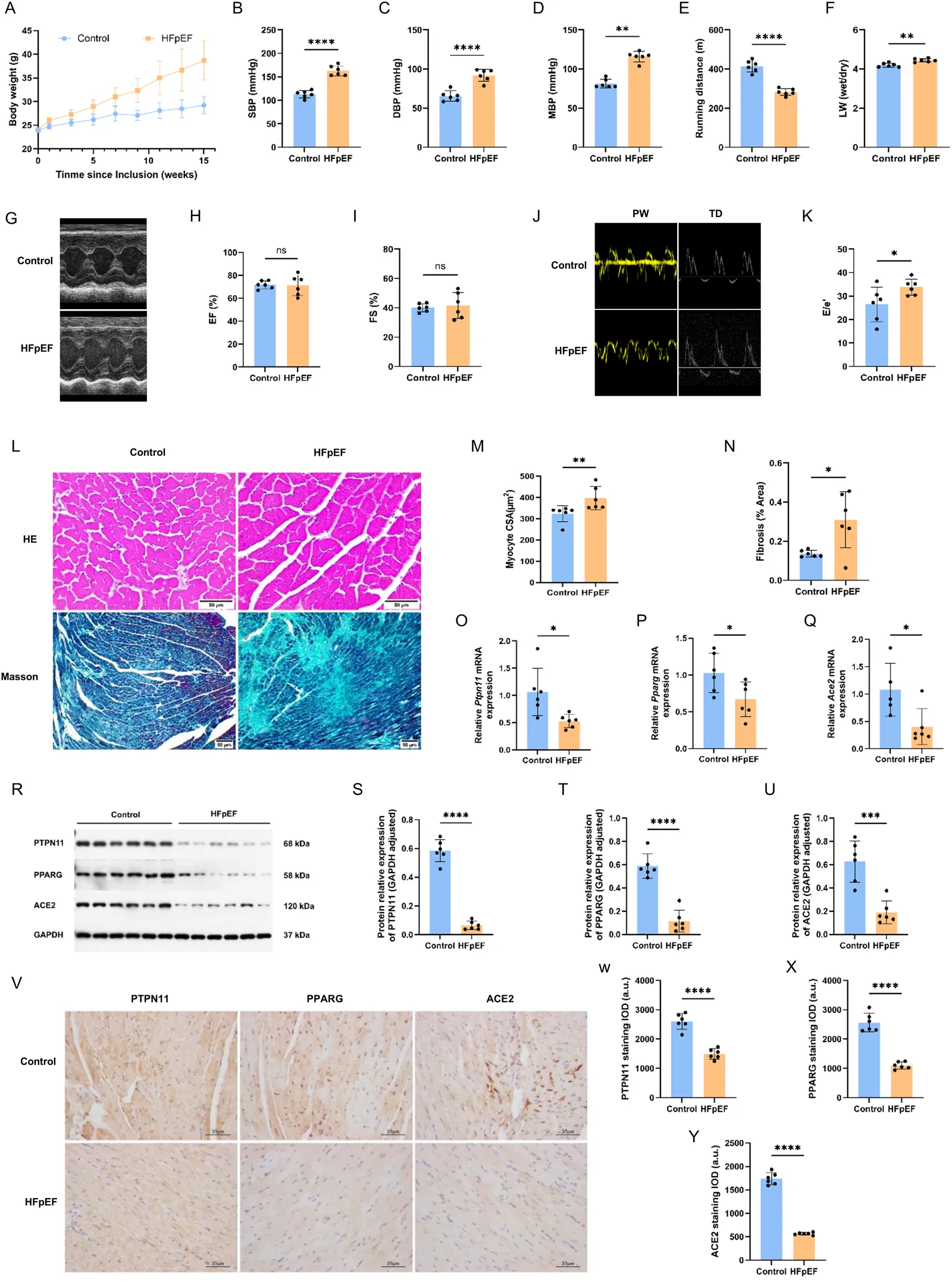
Establishment of the HFD/L-NAME-induced HFpEF mouse model and *in vivo* validation of hub PMFRDEGs. Unless otherwise stated, n = 6 animals per group, and each plotted biological replicate represents one animal. **(A)** Body-weight trajectory (g), analyzed by two-way repeated-measures ANOVA with time and group as factors and animal as the repeated factor; sphericity was not assumed, the Geisser–Greenhouse correction was applied, and control-versus-HFpEF comparisons at individual time points were adjusted using the Šídák procedure. **(B–D)** Systolic, diastolic, and mean arterial pressure (mmHg), respectively. **(E)** Treadmill running distance (m). **(F)** Lung wet-to-dry weight ratio (dimensionless). **(G)** Representative M-mode echocardiographic images. **(H, I)** LVEF and LVFS (%). **(J)** Representative pulsed-wave and tissue-Doppler images. **(K)** E/e′ ratio (dimensionless). **(L)** Representative H&E- and Masson-stained myocardial sections. **(M)** Cardiomyocyte cross-sectional area (μm²). **(N)** Fibrotic area (%). **(O–Q)** Relative myocardial mRNA abundance of *Ptpn11*, *Pparg*, and *Ace2*, respectively, expressed as 2^−ΔΔ^*^Ct^* relative values. **(R)** Representative Western blots of PTPN11, PPARG, and ACE2, with GAPDH as the loading control; full uncropped blots are provided in **Supplementary Figure S1**. **(S–U)** GAPDH-adjusted densitometric ratios for PTPN11, PPARG, and ACE2, respectively (dimensionless). **(V)** Representative immunohistochemical staining for PTPN11, PPARG, and ACE2. **(W–Y)** Animal-level IOD values for PTPN11, PPARG, and ACE2, respectively (a.u.). Two-sided unpaired Student’s t-tests were used for panels **(B, C, E, F, H, K, P, S–U, W, X)** two-sided Welch’s t-tests were used for panels **(I, N, O, Y)** and two-sided Mann–Whitney U tests were used for panels **(D, M)**, and **(Q)** No multiplicity adjustment was applied to these single-time-point two-group comparisons; their *P* values are nominal and two-sided. For *Ace2* mRNA in panel **(Q)** n = 5 in the control group and n = 6 in the HFpEF group because one control measurement was not quantifiable and the missing value was not imputed. For panel **(A)** asterisks denote Šídák-adjusted *P* values; for all other quantitative panels, asterisks denote nominal two-sided *P* values. ns, *P* ≥ 0.05; ^∗^*P* < 0.05; ^∗∗^*P* < 0.01; ^∗∗∗^*P* < 0.001; ^∗∗∗∗^*P* < 0.0001.

Except for the Šídák-adjusted between-group comparisons at individual time points in the longitudinal body-weight analysis (**Figure 2A**), no multiplicity adjustment was applied to the descriptive between-group comparisons in **Table 2** or to the single-time-point two-group comparisons in the murine validation experiments; the corresponding *P* values are therefore nominal and two-sided. For the exploratory clinical whole-blood analysis, *PTPN11* mRNA abundance was compared between patients with HFpEF and control participants using a two-sided Mann–Whitney U test. The ability of whole-blood *PTPN11* mRNA abundance to distinguish HFpEF from control status was explored using ROC curve analysis in GraphPad Prism. HFpEF was treated as the positive clinical state and the control group as the reference state, with the classification direction oriented such that lower *PTPN11* abundance corresponded to HFpEF. Discriminatory performance was summarized by the area under the ROC curve (AUC) and its 95% confidence interval. Sensitivity and specificity were expressed as percentages, with their 95% confidence intervals calculated using the Wilson–Brown method implemented in Prism. Given the modest size of the clinical cohort, the ROC analysis was interpreted as exploratory. Associations of whole-blood *PTPN11* mRNA abundance with NT-proBNP and C-reactive protein (CRP) within the HFpEF group were quantified using two-sided Spearman’s rank correlation coefficients with 95% confidence intervals. Where shown, simple linear-regression lines with 95% confidence bands were superimposed on the scatter plots only to illustrate the direction of the data trend; inferential conclusions for these associations were based on Spearman’s rank correlation rather than on the regression slope.

Unless otherwise specified, all statistical tests were two-sided, and *P* < 0.05 was considered statistically significant. Numerical *P* values are reported where available; where graphical significance symbols are used, the corresponding *P* value thresholds and whether the values are nominal or multiplicity-adjusted are defined in the relevant figure legends.

## 3 Results

### 3.1 Technology roadmap

**Figure 3** illustrates the overall workflow for the identification of pyroptosis- and myocardial fibrosis-related candidate genes and their subsequent downstream validation in HFpEF.

**Figure 3:**
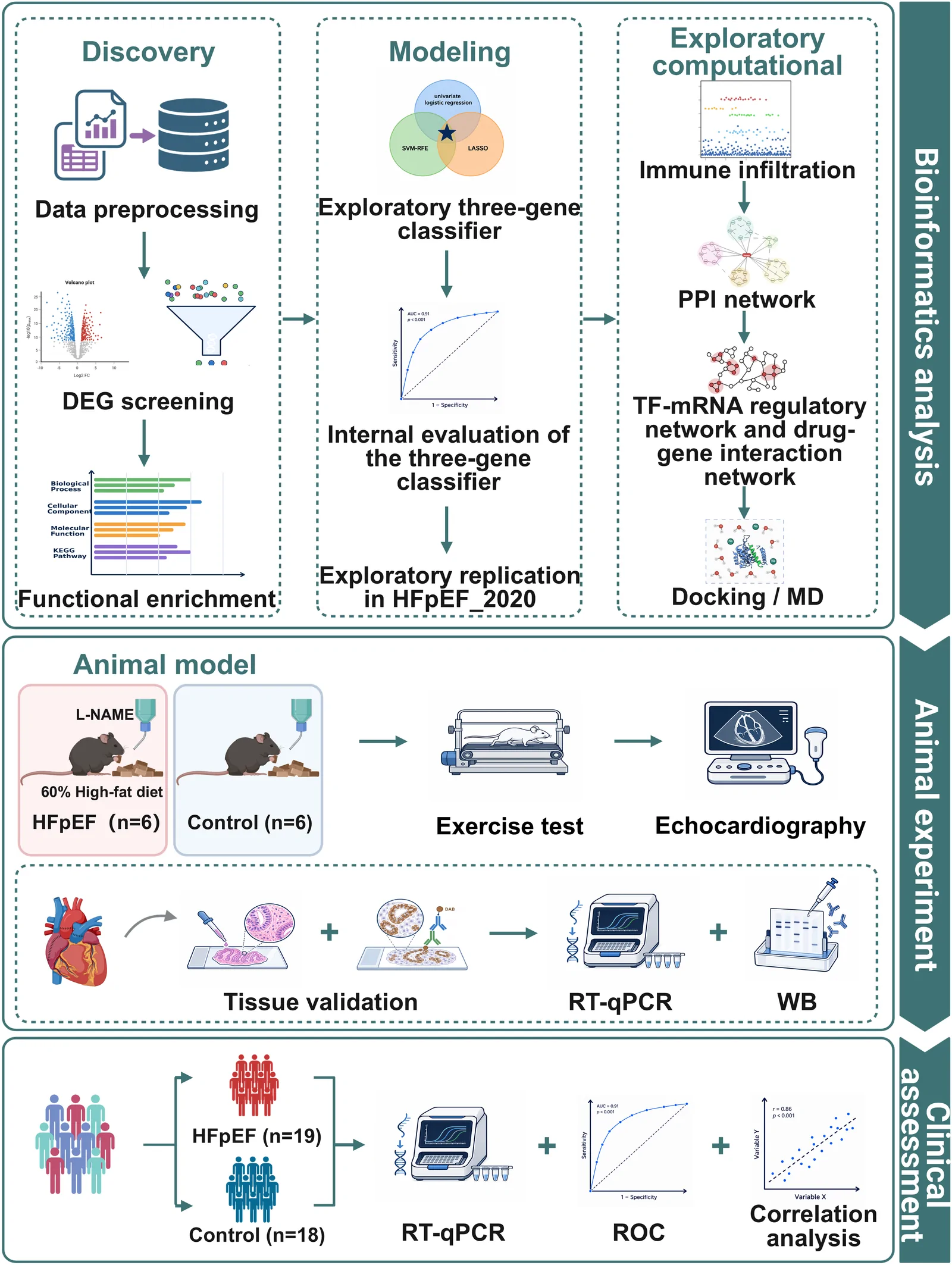
Study workflow for the identification and downstream evaluation of pyroptosis- and myocardial fibrosis-related candidate genes in HFpEF. The diagram summarizes transcriptomic data acquisition and preprocessing, exploratory differential-expression screening, integration with the predefined pyroptosis- and myocardial fibrosis-related gene sets, functional enrichment, feature selection, classifier construction, immune-cell deconvolution, network and structural analyses, and subsequent murine and clinical expression studies. Biological sample size, inferential statistical testing, multiplicity adjustment, physical measurement units, and asterisk notation are not applicable to this schematic workflow.

### 3.2 Integration of heart failure datasets with preserved ejection fraction

To construct the integrated GEO cohort, batch effects between GSE180065 and GSE194151 were corrected using the R package sva. Boxplots of expression-profile distributions before and after correction showed improved consistency across datasets (**Figures 4A, B**). Both datasets contained HFpEF and control samples, so disease status was not completely confounded with dataset source. Nevertheless, the unequal HFpEF-to-control ratios (10:5 in GSE180065 and 15:15 in GSE194151) created partial design imbalance. PCA showed less dataset-associated clustering after ComBat adjustment (**Figures 4C, D**). This pattern supports attenuation of dataset-associated technical structure but does not demonstrate that the partial association between dataset source and disease status, or biological heterogeneity arising from differences in induction protocol, treatment duration, or sex composition, was eliminated.

**Figure 4:**
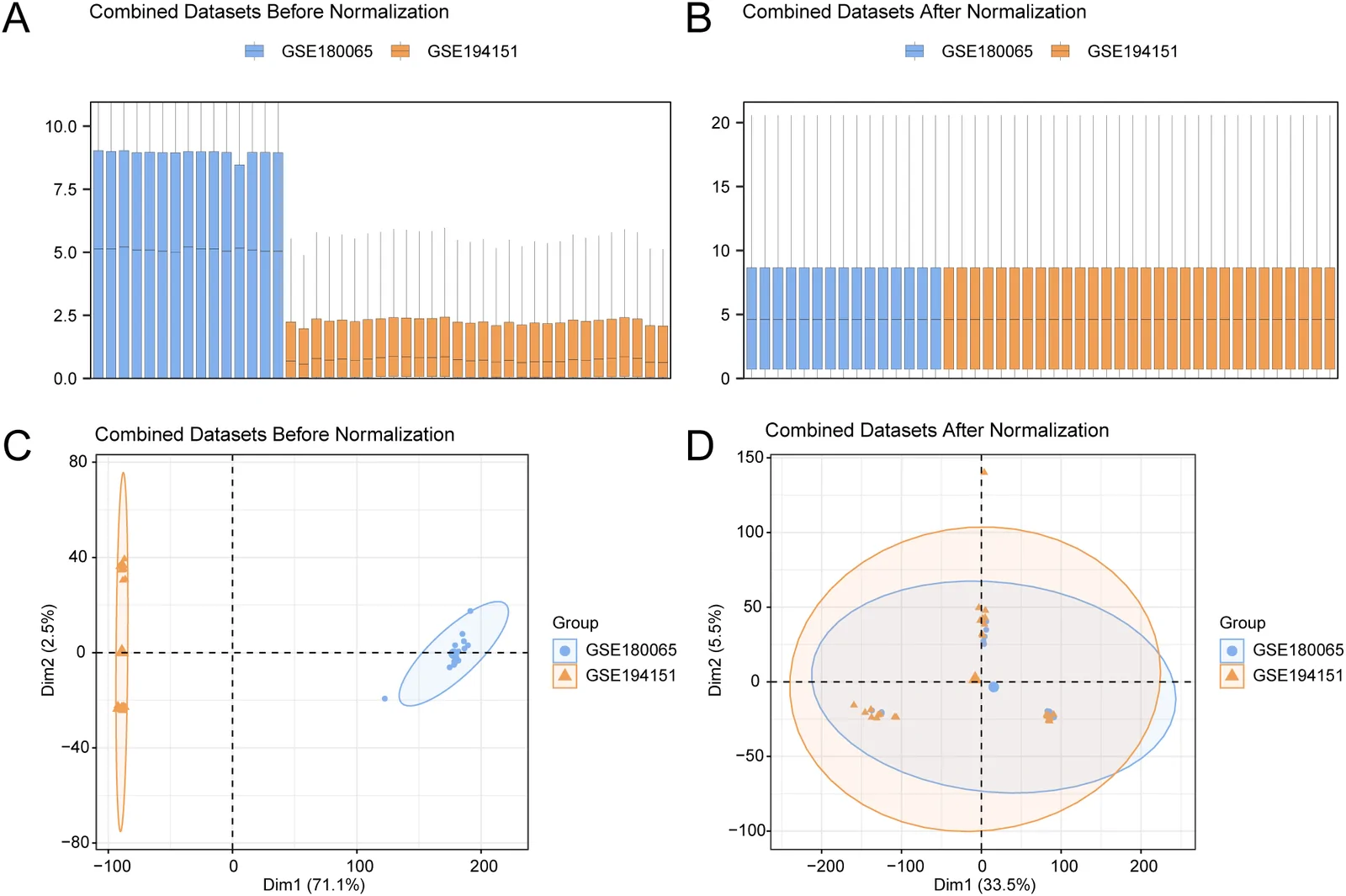
Batch-effect correction of GSE180065 and GSE194151. All 45 murine cardiac RNA-seq samples were retained in the integrated analysis (control, n = 20; HFpEF, n = 25). **(A, B)** Expression-profile distributions before and after batch correction, respectively; expression values are shown on the normalized log2(FPKM + 1) scale. **(C, D)** Principal-component analysis before and after batch correction; PCA scores are dimensionless. These panels are descriptive and were used to inspect dataset-associated structure rather than to test a prespecified biological hypothesis. Inferential statistical testing and multiplicity adjustment are therefore not applicable, and asterisk notation is not used.

### 3.3 Identification of pyroptosis- and myocardial fibrosis-associated DEGs in HFpEF

Differential expression analysis was subsequently carried out between HFpEF and control samples using the limma package. Under the exploratory criterion (|log_2_FC| > 0, nominal *P* < 0.05), 2,294 genes entered the candidate pool, of which 1,051 had positive and 1,243 had negative log_2_FC values (**Figure 5A**). Intersection with the predefined PMFRG set yielded six candidates—*Pparg*, *Ace2*, *Smad2*, *Lcn2*, *Ptpn11*, and *Sqstm1* (**Figure 5B**).

**Figure 5:**
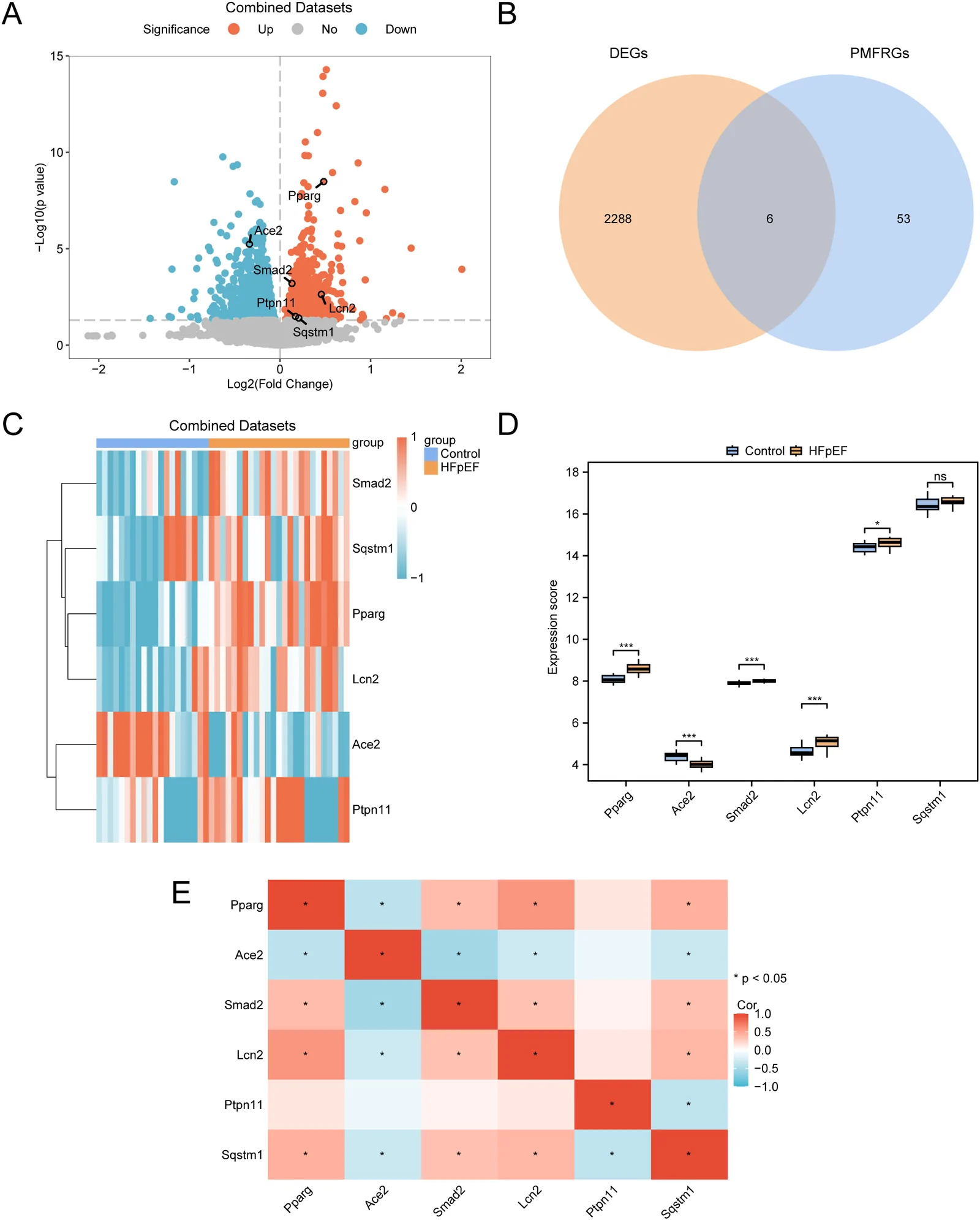
Differential-expression analysis in the integrated murine discovery cohort (control, n = 20; HFpEF, n = 25; total n = 45). **(A)** Volcano plot derived from limma empirical-Bayes differential-expression modeling of normalized log2(FPKM + 1) expression data. The primary exploratory screen used nominal *P* < 0.05 without multiplicity adjustment; Benjamini–Hochberg FDR analyses were performed separately as sensitivity analyses. **(B)** Intersection of the exploratory candidate pool with the predefined PMFRG set; no independent statistical test applies to this Venn diagram. **(C)** Heatmap of the six resulting PMFRDEGs; no additional inferential test applies. **(D)** Between-group expression of the PMFRDEGs; statistical annotations correspond to the limma differential-expression analysis. **(E)** Pairwise relationships among PMFRDEGs quantified by Spearman’s rank correlation; no multiplicity adjustment was applied to these exploratory correlations. Expression values, log_2_FC, and Spearman’s *ρ* are dimensionless. Where statistical symbols are displayed, they denote nominal *P* values: ns, *P* ≥ 0.05; ^∗^*P* < 0.05; ^∗∗^*P* < 0.01; ^∗∗∗^*P* < 0.001; ^∗∗∗∗^*P* < 0.0001.

The candidate set contracted substantially under multiplicity control. When Benjamini–Hochberg FDR < 0.05 was combined with |log_2_FC| > 0.5, 47 DEGs remained and none overlapped the PMFRG set. When FDR < 0.05 was applied without an effect-size cutoff, 315 DEGs remained, with *Ace2*, *Pparg*, and *Smad2* forming the PMFRDEG intersection. The original six-gene set is therefore threshold-sensitive and is carried forward only as an exploratory screening pool. Analysis of GSE194151 separately, including sex-stratified sensitivity analyses, did not stably reproduce the PMFRDEG pattern obtained from the merged cohort. This discordance was considered evidence of cohort- and subgroup-dependence rather than confirmation of a fixed six-gene signature. Their expression patterns across HFpEF and control samples were visualized using the pheatmap package (**Figure 5C**), and between-group differences are shown in **Figure 5D**. Significant expression differences were observed for *Pparg*, *Ace2*, *Smad2*, and *Lcn2* (*P* < 0.001), while *Ptpn11* also differed significantly between groups (*P* < 0.05). In the integrated GEO cohort, correlation analysis revealed that *Pparg*, *Smad2*, *Lcn2*, and *Sqstm1* tended to vary in the same direction, showing broadly positive interrelationships. By contrast, *Ace2* and *Ptpn11* were inversely associated with several other PMFRDEGs (**Figure 5E**).

### 3.4 Functional characterization of PMFRDEGs

GO and KEGG analyses were used to probe candidate functional annotations for the six PMFRDEGs, rather than to assign established biological roles. In the GO biological process domain, the six-gene set mapped most conspicuously to annotations involving transmembrane transport, regulation of receptor-mediated signaling, and peptide hormone secretion. The cellular component output was somewhat more variegated, encompassing the RNA polymerase II transcription regulator complex, SMAD protein complex, amphisome, small ubiquitin-like modifier (SUMO) ligase complex, and autolysosome. Within the molecular-function domain, the most conspicuous annotation signals involved receptor-regulated SMAD (R-SMAD) binding, SMAD binding, signaling adaptor activity, phosphatase binding, and ubiquitin protein ligase binding; with only six input genes, the relative prominence of any single term remains sensitive to individual gene contributions.

KEGG returned several candidate pathway labels—including osteoclast differentiation, efferocytosis, cellular senescence, proteoglycans in cancer, and the renin-angiotensin system—but this compact input set does not support an inference that any of these pathways is globally activated, suppressed, or mechanistically dominant in HFpEF. The full set of enrichment results is provided in **Supplementary Table S4**, while the distribution of the annotated terms is displayed in **Figure 6A**. To further map how individual genes were connected with these functional annotations, term-gene networks were constructed from the GO and KEGG enrichment outputs. These networks summarize the biological process, cellular component, molecular function, and pathway categories shown in **Figures 6B–E**. In each network, node size reflects the number of PMFRDEGs linked to a given term.

**Figure 6:**
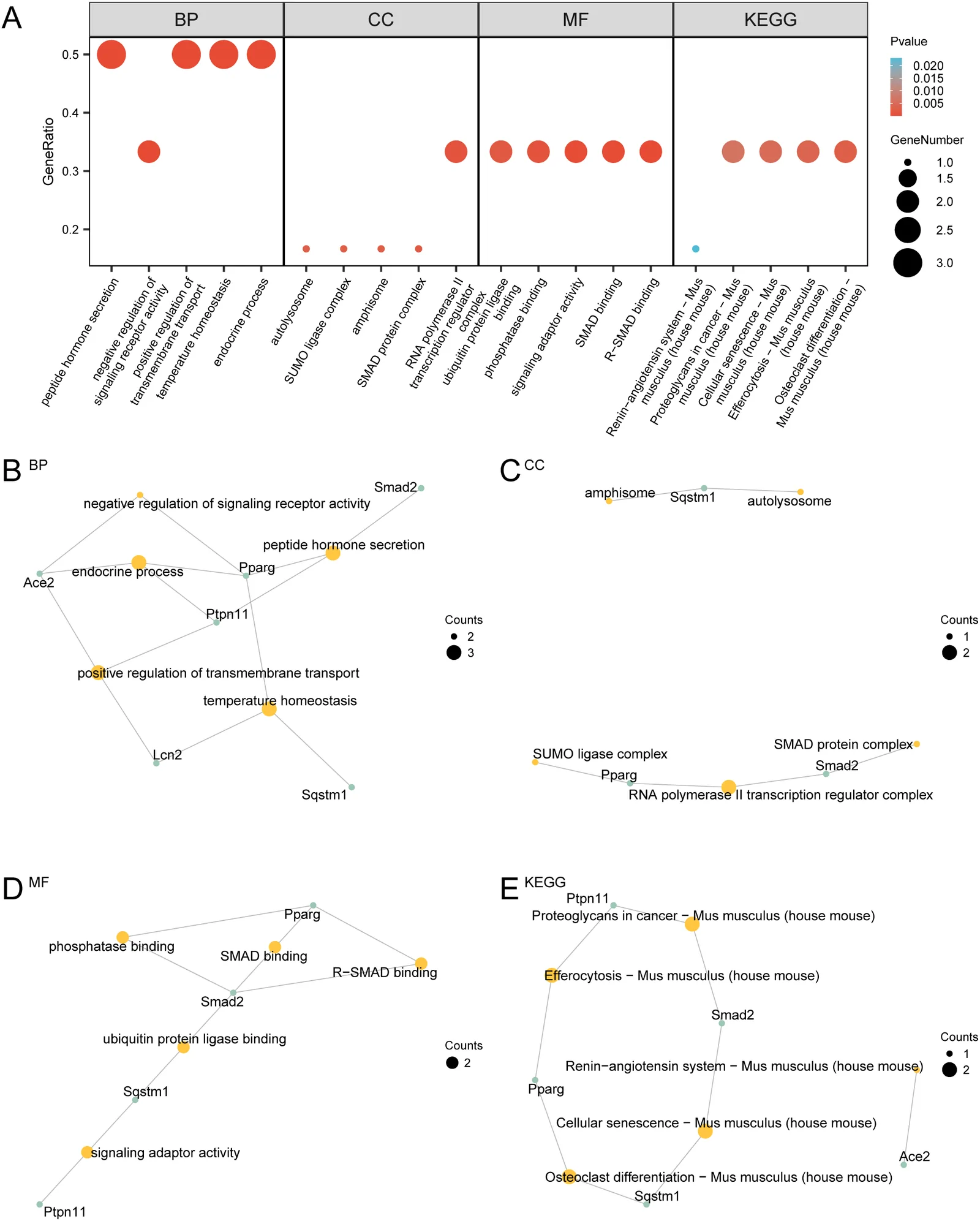
GO and KEGG enrichment analyses of the six PMFRDEGs. **(A)** Bubble plot of the GO and KEGG enrichment output. **(B–E)** Term–gene networks depicting the corresponding biological-process, cellular-component, molecular-function, and KEGG annotations. Enrichment analyses were performed with clusterProfiler, using all genes retained after preprocessing and annotation as the background; *P* values were adjusted using the Benjamini–Hochberg procedure, and terms with raw *P* < 0.05 and qvalue < 0.25 were retained for exploratory interpretation. Biological n is not applicable to these enrichment panels; the analytical input comprised six PMFRDEGs derived from the 45-sample discovery cohort. GeneRatio and FDR are dimensionless. Asterisk notation is not used.

### 3.5 Exploratory feature selection and classifier construction

Each of the six PMFRDEGs was first examined in a separate univariate logistic regression. *Ace2*, *Lcn2*, *Pparg*, *Ptpn11*, and *Smad2* met the *P* < 0.05 screening criterion and were advanced to the subsequent SVM-RFE analysis. **Figure 1A** presents the corresponding *β* coefficients with Wald 95% confidence intervals rather than odds ratios. These five genes were subsequently examined using the 10-fold SVM-RFE procedure described above. Across the evaluated feature-set sizes, the lowest mean held-out classification error was obtained with a three-feature subset (**Figures 1B, C**). On the basis of the aggregated feature ranking, *Ace2*, *Pparg*, and *Ptpn11* were retained for subsequent LASSO analysis (**Figure 1D**). The held-out classification error used to select the three-feature subset pertains to the SVM-RFE subset-evaluation stage and should not be interpreted as a nested validation estimate of the complete univariate logistic regression–SVM-RFE–LASSO pipeline. LASSO regression retained *Ace2*, *Pparg*, and *Ptpn11* at lambda.min, and these genes were therefore taken forward as the fixed three-gene panel (**Figures 1E, F**). Their selection was far less uniform when the downstream feature-selection procedure was repeated within nested resampling: across 250 outer training folds, selection frequencies were 100.0% for *Pparg*, 89.2% for *Ace2*, 52.8% for *Smad2*, 44.8% for *Lcn2*, 38.4% for *Ptpn11*, and 3.6% for *Sqstm1*. *Pparg*, *Ptpn11*, and *Ace2* entered the final model together in 34.0% of outer folds, whereas the exact three-gene combination was recovered in only 13.2%, exposing appreciable selection uncertainty for *Ptpn11* and for the panel as a whole.

### 3.6 Evaluation of the exploratory HFpEF classifier

Formal separation detection confirmed complete separation for the ordinary three-gene logistic model HFpEF ~ *Pparg* + *Ptpn11* + *Ace2*; the maximum-likelihood fit did not converge and produced numerically extreme fitted probabilities. The final model was therefore refitted using Firth bias-reduced logistic regression. The resulting coefficients for *Pparg*, *Ptpn11*, and *Ace2* were 15.642, 10.659, and −11.302, respectively, corresponding to odds ratios of 6.21 × 10^6^ (95% CI, 17.10–2.26 × 10^12^; *P* = 3.56 × 10^-6^), 4.26 × 10^4^ (95% CI, 2.26–8.00 × 10^8^; *P* = 0.00298), and 1.24 × 10^-5^ (95% CI, 6.29 × 10^-10^–0.242; *P* = 0.00310). The overall penalized likelihood-ratio test yielded *P* = 4.29 × 10^-10^. Because the predictors were not Z-score standardized, each odds ratio refers to a one-unit increase on the preprocessed log2 expression scale. The Firth model had an apparent AUC of 1.0000 and an apparent Brier score of 0.01058. Of the 2,000 stratified bootstrap resamples, 1,997 completed successfully. The mean optimism in AUC was 0.00294, yielding an optimism-corrected AUC of 0.9971 (95% bootstrap percentile interval, 0.9780–1.0000); the corresponding optimism-corrected Brier score was 0.02505 (0.00853–0.06409) (**Figure 7A**). The apparent calibration intercept and slope were 0.1565 and 1.6597, respectively. After optimism correction, the calibration intercept was 0.2428 (95% bootstrap percentile interval, −0.7402–1.2763) and the calibration slope was 1.6710 (0.8315–3.1674). Because calibration was estimated with Firth auxiliary models rather than ordinary maximum-likelihood logistic regression, the apparent intercept and slope are not mathematically constrained to exactly 0 and 1 (**Figure 7B**). Repeated nested cross-validation yielded a more conservative estimate of model-development performance. All 250 outer folds completed successfully; across 50 repeats, the mean out-of-fold AUC was 0.9286 (median, 0.9300; SD, 0.0245; 2.5%–97.5% percentile interval, 0.8726–0.9700), and the mean Brier score was 0.1026 (median, 0.1034; 2.5%–97.5% percentile interval, 0.0696–0.1560). The lower out-of-fold performance relative to the fixed-panel bootstrap estimate reflects the additional uncertainty introduced when downstream feature selection and model fitting are repeated within the resampling process. Within the same discovery cohort, decision-curve analysis based on the Firth-model predictions yielded a higher estimated net benefit than the treat-all and treat-none reference strategies across part of the examined threshold range (**Figure 7C**). Because this analysis remained internal to the model-development cohort, the result was interpreted as descriptive rather than as evidence of established clinical utility. The nomogram in **Figure 7D** was reconstructed from the finite Firth coefficients and is presented only as a graphical representation of the fitted discovery model, not as a transferable clinical scoring system.

**Figure 7:**
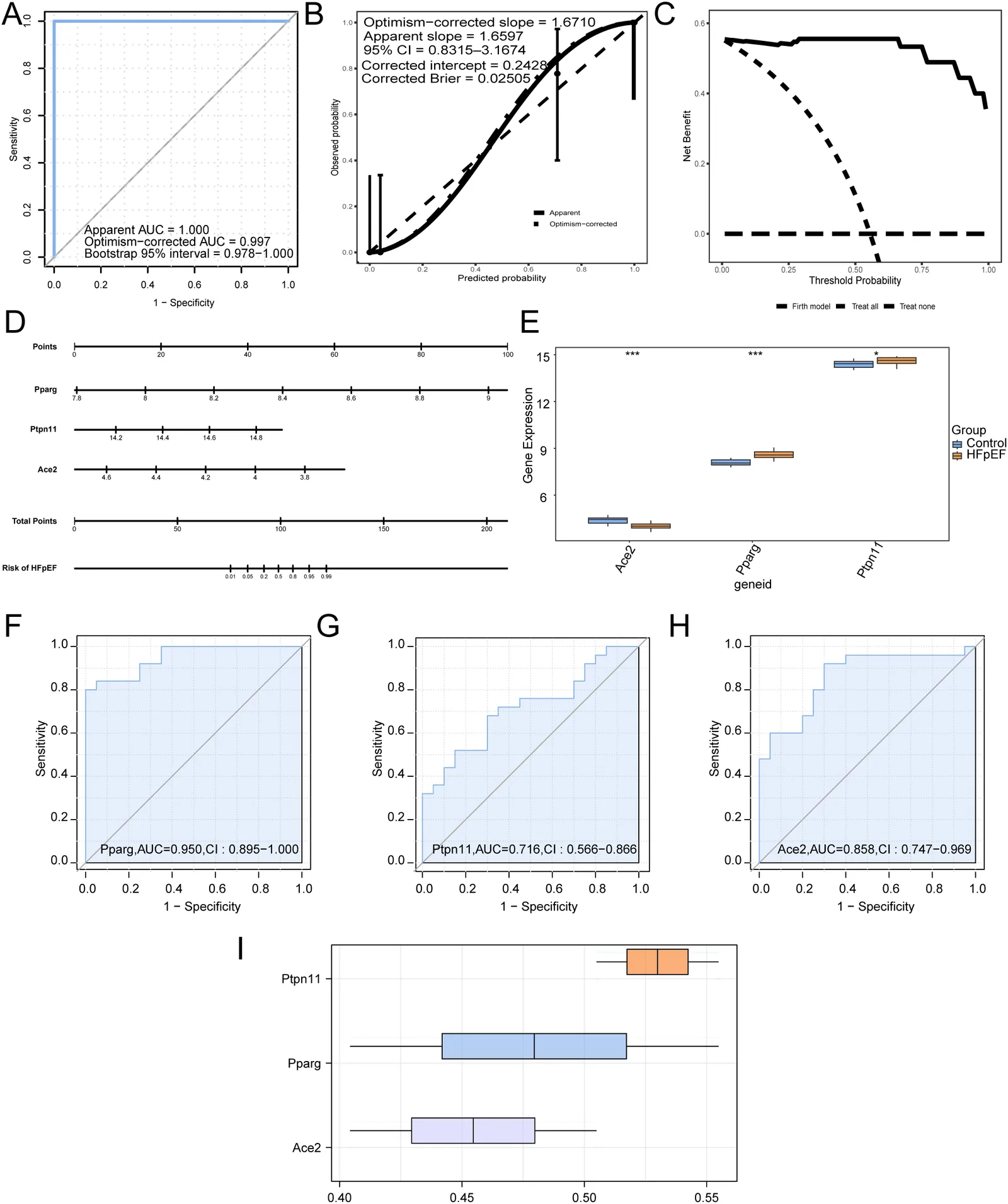
Internal evaluation of the fixed three-gene Firth classifier in the merged murine discovery cohort (control, n = 20; HFpEF, n = 25; total n = 45). **(A)** Apparent ROC curve of the Firth model (AUC = 1.0000); 2,000 stratified bootstrap resamples yielded an optimism-corrected AUC of 0.9971 (95% bootstrap percentile interval, 0.9780–1.0000). **(B)** Firth-based calibration analysis; the optimism-corrected calibration intercept was 0.2428 (95% bootstrap percentile interval, −0.7402–1.2763), and the calibration slope was 1.6710 (0.8315–3.1674). **(C)** In-sample decision curve analysis based on Firth-model predictions. **(D)** Nomogram reconstructed from the fitted Firth coefficients. Repeated nested cross-validation results are reported separately in the Results and are not represented by panel **(A, E)** Normalized log2(FPKM + 1) expression of *Pparg*, *Ptpn11*, and *Ace2* in HFpEF and control samples; groups were compared using two-sided Wilcoxon rank-sum tests, with no multiplicity adjustment. **(F–H)** Individual ROC curves for *Pparg*, *Ptpn11*, and *Ace2*. **(I)** GO semantic-similarity analysis. In panel **E**, asterisks denote nominal two-sided *P* values: ns, *P* ≥ 0.05; ^∗^*P* < 0.05; ^∗∗^*P* < 0.01; ^∗∗∗^*P* < 0.001; ^∗∗∗∗^*P* < 0.0001. Asterisk notation is not used for panels **(A–D)** or **(F–I)**.

The three candidate diagnostic genes exhibited clearly divergent expression patterns between HFpEF and control samples (**Figure 7E**). The genes did not move in parallel. *Pparg* expression was increased in the HFpEF group, whereas *Ace2* was reduced; both between-group differences were pronounced (*P* < 0.001). *Ptpn11* also shifted upward in HFpEF, although the between-group evidence was more modest (*P* < 0.05). ROC analysis of each gene suggested that their diagnostic performance was not identical. *Ace2* and *Ptpn11* had moderate discriminative ability, with AUC values of 0.70–0.90, whereas *Pparg* showed a stronger diagnostic signal, with an AUC exceeding 0.90 (**Figures 7F–H**). Functional similarity analysis further highlighted *Ptpn11* as a candidate with potential biological relevance among the diagnostic genes, but this interpretation should still be regarded as preliminary until supported by experimental validation (**Figure 7I**).

### 3.7 Exploratory replication of the three-gene panel in HFpEF_2020

The HFpEF_2020 dataset comprised left-ventricular transcriptomic profiles from 41 patients with HFpEF and 45 controls (19). After Ensembl ortholog matching and expression-matrix preprocessing, *PPARG*, *PTPN11*, and *ACE2* were retained as the prespecified panel and a logistic model was re-estimated within HFpEF_2020. The resulting apparent AUC exceeded 0.90 (**Figure 8A**). Because the coefficients were estimated anew in this human dataset, the ROC, calibration, and decision-curve results shown in **Figures 8A–C** represent in-dataset summaries of panel-level replication rather than external validation of the locked murine discovery model.

**Figure 8:**
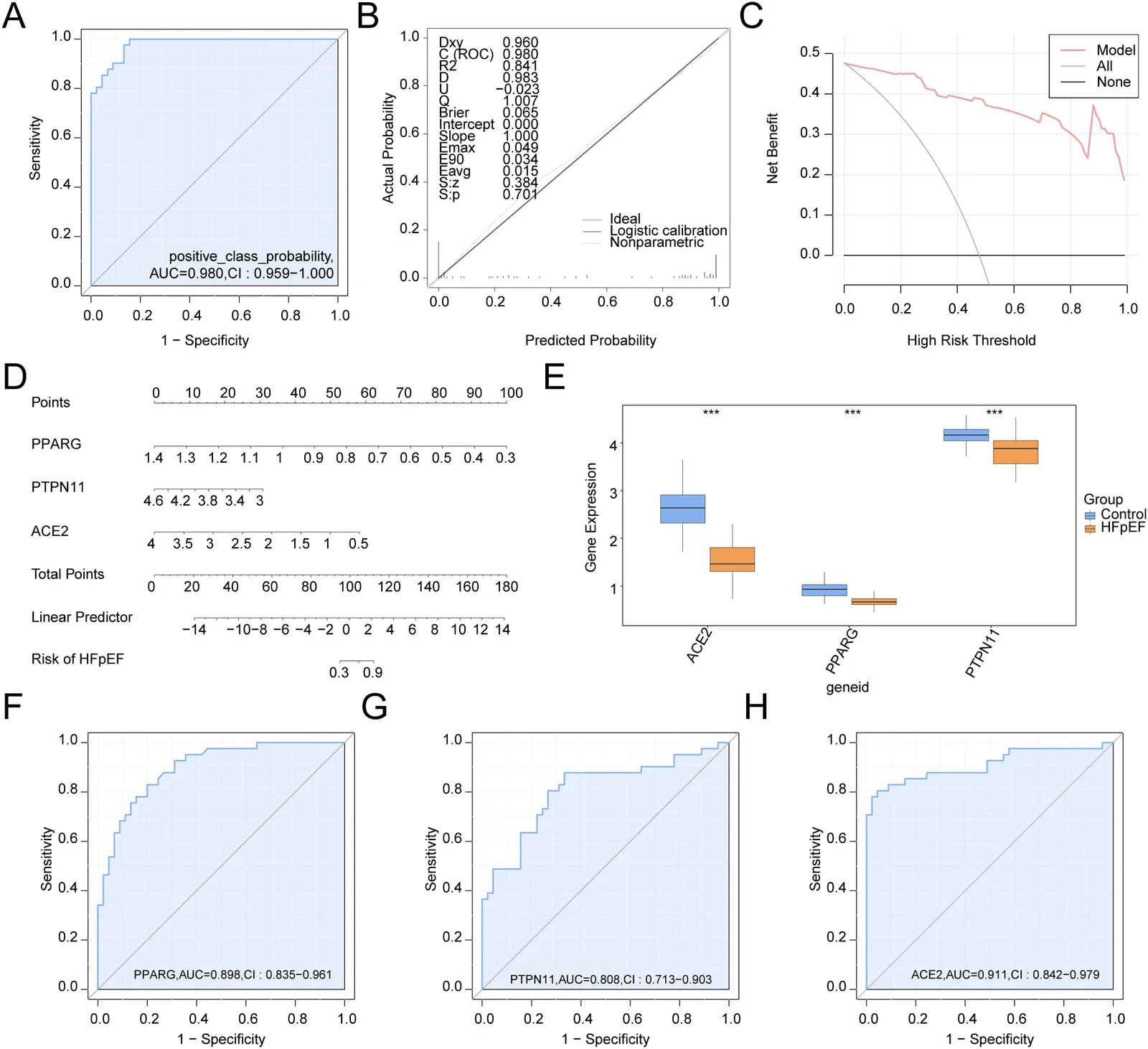
Exploratory replication of the three-gene panel in the human HFpEF_2020 left-ventricular dataset (control, n = 45; HFpEF, n = 41; total n = 86). The logistic model was re-estimated within HFpEF_2020 rather than evaluated using locked coefficients from the murine discovery cohort. **(A)** ROC curve of the refitted three-gene model. **(B, C)** Calibration and decision-curve analyses of the refitted model. **(D)** Dataset-specific nomogram generated from the HFpEF_2020 coefficients. **(E)** Normalized log2(FPKM + 1) expression of *PPARG*, *PTPN11*, and *ACE2* in HFpEF and control samples; groups were compared using two-sided Wilcoxon rank-sum tests, without multiplicity adjustment. **(F–H)** Individual ROC curves for *PPARG*, *PTPN11*, and *ACE2*. In panel **(E)**, asterisks denote nominal two-sided *P* values: ns, *P* ≥ 0.05; ^∗^*P* < 0.05; ^∗∗^*P* < 0.01; ^∗∗∗^*P* < 0.001; ^∗∗∗∗^*P* < 0.0001. Asterisk notation is not used for panels **(A–D)** or **(F–H)**.

The nomogram shown in **Figure 8D** was generated from coefficients re-estimated within HFpEF_2020 using *PPARG*, *PTPN11*, and *ACE2*. Its axis scales therefore reflect a dataset-specific human fit and should not be interpreted as the same locked coefficient model shown in **Figure 7D** or as a directly transferable cross-cohort risk score. In this scoring framework, *PPARG* carried a greater point contribution to the predicted risk than *PTPN11*. All three genes differed significantly between HFpEF and control samples in HFpEF_2020 (*P* < 0.001; **Figure 8E**). *PPARG* and *PTPN11* were lower in HFpEF, reversing the directions observed in the murine discovery cohort, whereas *ACE2* remained lower and therefore retained its discovery-stage direction. Individual ROC curve analysis showed that *ACE2* had the strongest discriminatory performance, with an AUC above 0.90. By comparison, *PPARG* and *PTPN11* demonstrated moderate discrimination, with AUC values between 0.70–0.90 (**Figures 8F–H**).

### 3.8 Immune infiltration analysis

CIBERSORT analysis included all 45 discovery samples and generated relative-fraction estimates for 25 murine immune-cell types using the ImmuCC reference matrix (**Figure 9A**). B Cells Memory and DC Actived had estimated fractions of zero in every sample; the remaining 23 cell types were therefore carried into groupwise and correlation analyses. No inferred cell fraction differed significantly between HFpEF and control after multiplicity correction. T Cells CD4 Naive had the smallest unadjusted *P* value (*P* = 0.02042; Cliff’s delta = 0.376, 95% CI, 0.084–0.644), but the association did not survive Benjamini–Hochberg correction (FDR = 0.46965); none of the 23 comparisons had FDR < 0.05. Complete groupwise results for all 23 nonzero cell types are provided in **Supplementary Table S7**. Among the inferred fractions, M0 and M2 macrophages showed a strong inverse correlation (Spearman’s *ρ* = −0.890, *P* = 2.83 × 10^-16^; **Figure 9B**), whereas *Ptpn11* expression correlated positively with the inferred Mast Cells fraction (*ρ* = 0.525, *P* = 2.14 × 10^-4^; **Figure 9C**). These correlations describe patterns within compositional deconvolution estimates and were not interpreted as direct evidence of altered cell abundance, macrophage polarization, or mast-cell involvement.

**Figure 9:**
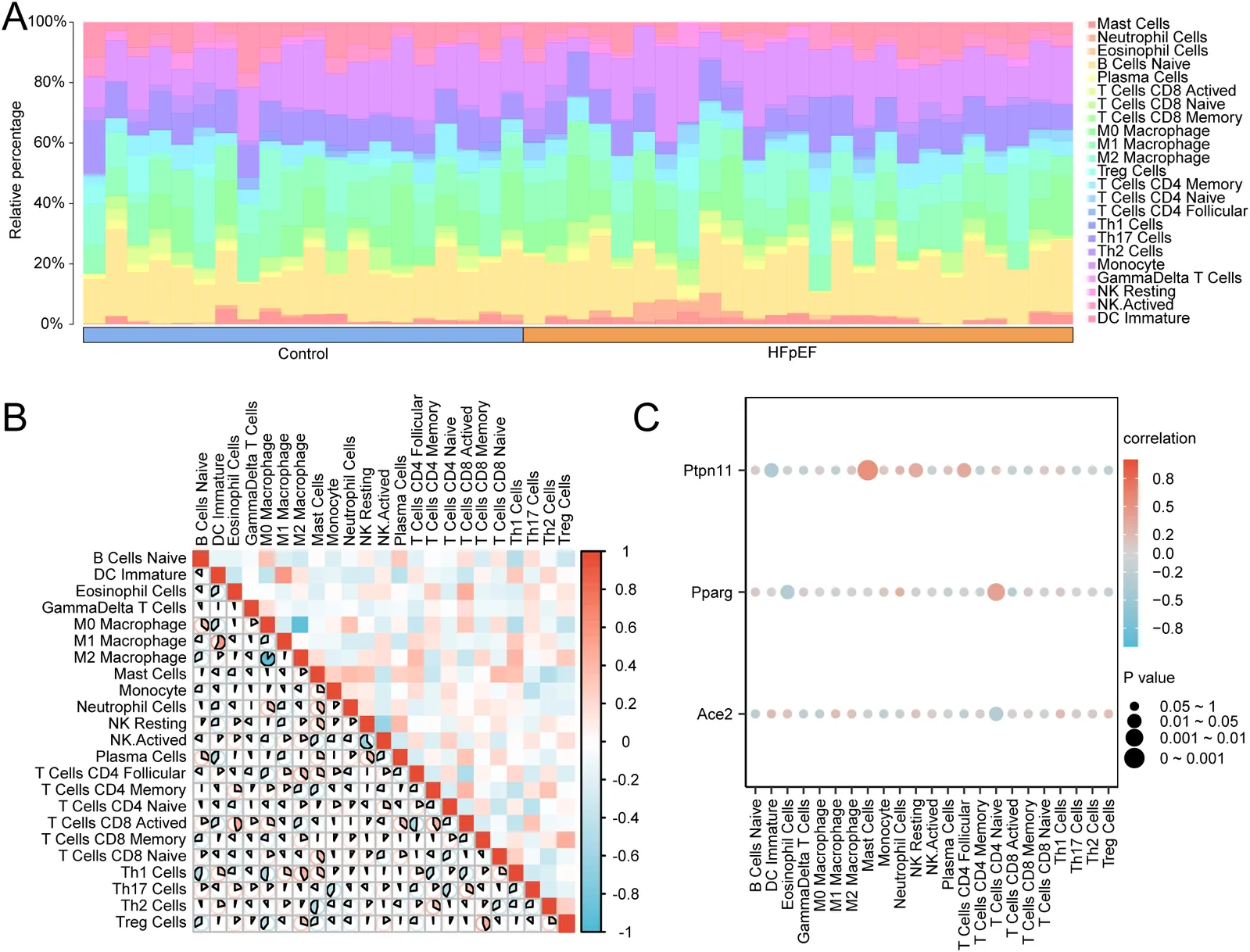
CIBERSORT-based immune-cell deconvolution in the integrated murine GEO cohort (control, n = 20; HFpEF, n = 25; total n = 45). **(A)** Relative immune-cell fractions across individual myocardial samples; fractions are dimensionless proportions, and no separate inferential test is attached to this compositional bar plot. **(B)** Pairwise associations among inferred immune-cell fractions quantified using Spearman’s rank correlation. **(C)** Associations between model-gene expression and inferred immune-cell fractions quantified using two-sided Spearman’s rank correlation; bubble size denotes the *P* value stratum (*P* ≤ 0.001, 0.001 < *P* ≤ 0.01, 0.01 < *P* ≤ 0.05, or *P* > 0.05). Spearman’s *ρ* and immune-cell fractions are dimensionless. No multiplicity adjustment was applied to the exploratory correlation analyses in panels **(B, C)**. Asterisk notation is not used; inferential statistical testing is not applicable to panel **(A)**.

### 3.9 Protein–protein interaction network and PPARG–PTPN11 docking analysis

We first used STRING to analyze protein–protein interaction networks involving the three candidate genes, *Ace2*, *Pparg*, and *Ptpn11* (**Figure 10A**). In the STRING-derived interaction network, ACE2, PPARG, and PTPN11 were positioned near the network core, suggesting that these proteins may serve as potential hubs. We then queried GeneMANIA to extend the functional association analysis and identify additional related proteins or genes. The final network comprised 20 proteins or genes with functional associations. Within this interaction map, colored edges marked co-expression relationships as well as shared protein-domain connections (**Figure 10B**).

**Figure 10:**
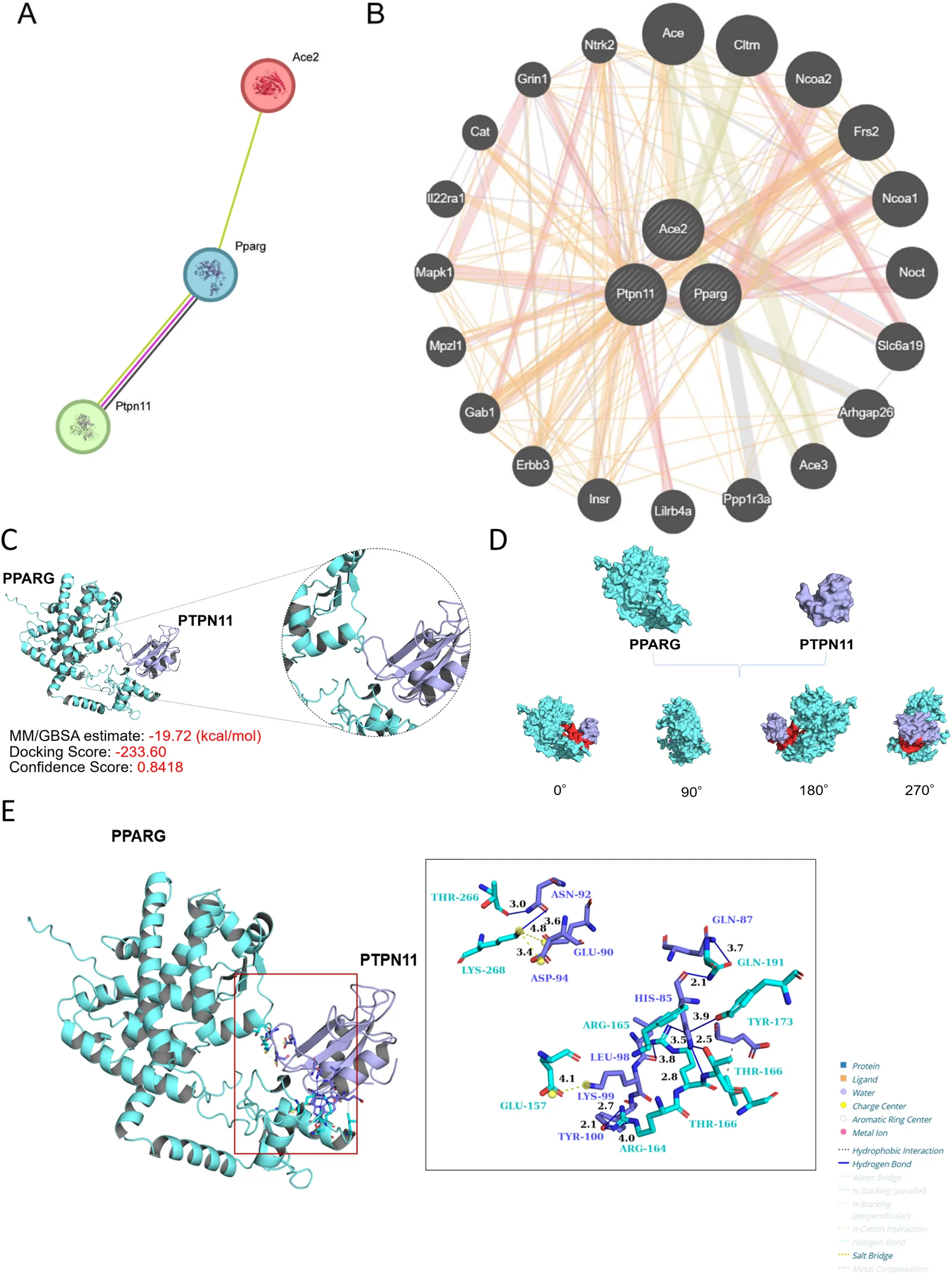
Protein–protein interaction networks and exploratory PPARG–PTPN11 docking analysis. **(A)** Protein-association network of the three candidate genes generated using STRING. **(B)** Functional-association network generated using GeneMANIA. **(C)** Top-ranked HDOCKlite v1.1 docking pose between full-length PPARG and the PTPN11 structural construct comprising residues 4–103. **(D)** Surface representation of the retained modeled complex. **(E)** Putative residue-level contacts at the modeled PPARG–PTPN11 interface annotated using PLIP. Biological n is not applicable because these panels represent database-derived and computational structural analyses. No inferential statistical test or multiplicity adjustment was performed, and asterisk notation is not used. HDOCK docking and confidence scores are model-ranking and docking-confidence metrics rather than thermodynamic binding free energies.

Protein–protein docking between full-length PPARG and the PTPN11 structural construct comprising residues 4–103 was performed using HDOCKlite v1.1 (**Figure 10C**). Among 100 generated candidate poses, model_1 was the top-ranked docking pose, with an HDOCK docking score of −233.60 and a confidence score of 0.8418. These values were interpreted as HDOCK ranking and confidence metrics rather than thermodynamic binding free energies. The retained docking model is shown in **Figure 10D**.

A separate MM/GBSA calculation performed using the default HawkDock protocol yielded a ΔG**_MM/GBSA_** estimate of −19.72 kcal/mol for the retained modeled PPARG–PTPN11 complex. No separate configurational-entropy term was estimated in this calculation. This MM/GBSA-derived energetic estimate was considered separately from the HDOCK docking score and confidence score.

The retained docking pose was subsequently analyzed using PLIP, which annotated several putative residue-level contacts at the modeled PPARG–PTPN11 interface (**Figure 10E**). Within the retained docking model, PLIP assigned a putative hydrogen-bond contact between Asn92 of PTPN11 and Thr266 of PPARG. Lys268 of PPARG was predicted to form salt-bridge contacts with Glu90 and Asp94 of PTPN11, whereas Gln191 of PPARG showed putative hydrogen-bond contacts with Gln87 and His85 of PTPN11. His85 of PTPN11 also showed predicted contacts with Tyr173, Thr166, and Arg165 of PPARG. Additional putative hydrogen-bond contacts involved Leu98 of PTPN11 and Arg165 of PPARG, and Tyr100 of PTPN11 and Arg164 of PPARG. A further putative salt bridge involved Lys99 of PTPN11 and Glu157 of PPARG.

### 3.10 Molecular dynamics trajectory analysis of the modeled PPARG–PTPN11 complex

Three independent 100-ns trajectories were analyzed for the modeled complex comprising full-length PPARG (chain A) and the PTPN11 structural construct comprising residues 4–103 (chain B). Overall backbone RMSD was used as a descriptor of conformational displacement from the starting coordinates; smaller RMSD values indicate less conformational drift within the sampled trajectory and should not be interpreted as evidence of stronger physiological binding. Across the three replicates, backbone RMSD reached a comparatively steady range after approximately 20 ns and subsequently fluctuated mainly between 0.5 and 0.8 nm (**Figure 11A**), without a sustained excursion in this global metric.

**Figure 11:**
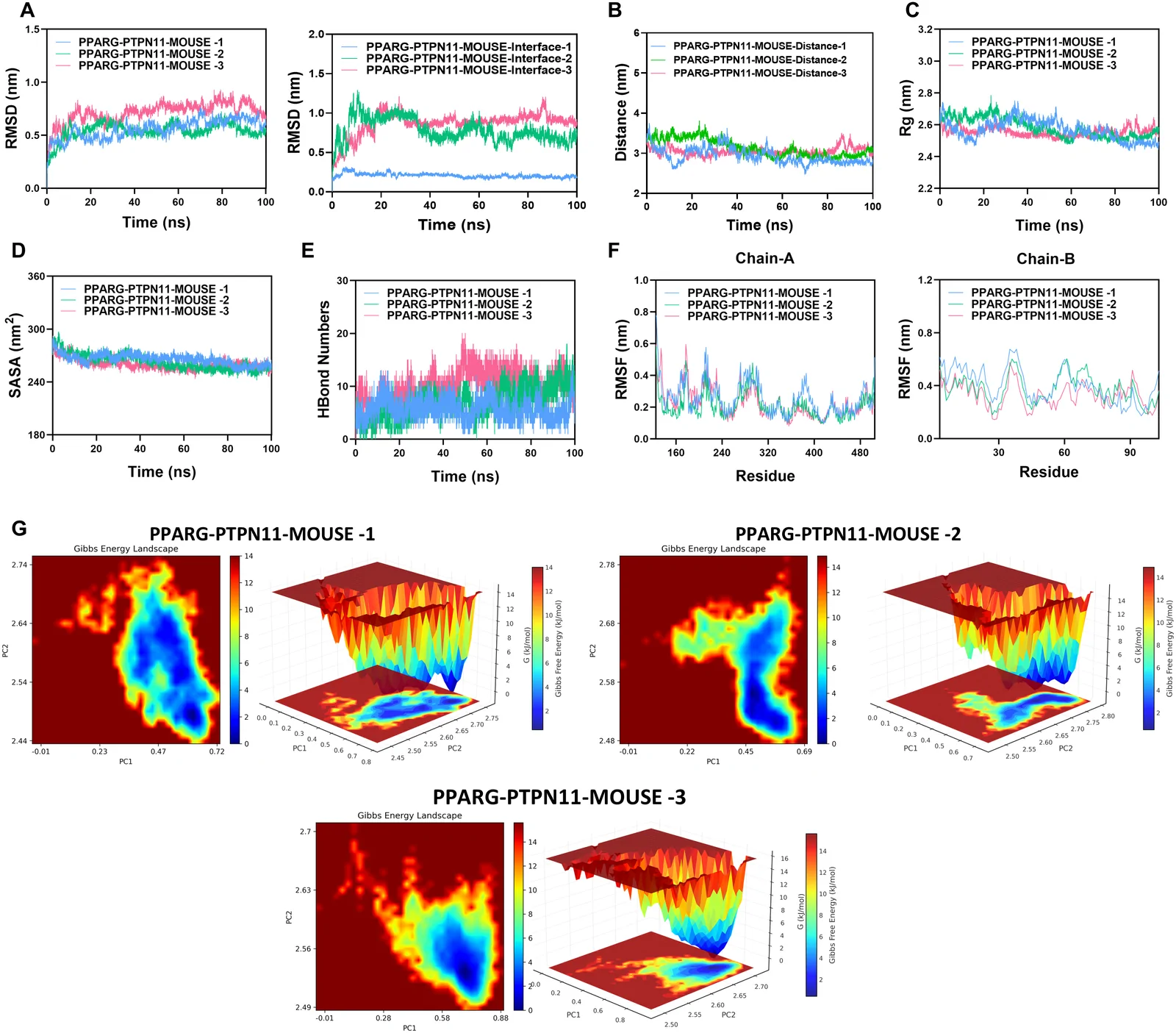
Molecular dynamics trajectory analysis of three independent 100-ns simulations of the modeled PPARG–PTPN11 complex. The modeled complex comprised full-length PPARG and a PTPN11 structural construct encompassing residues 4–103. **(A)** Overall backbone and interface RMSD. **(B)** Center-of-mass separation between the modeled chains. **(C)** Radius of gyration. **(D)** Solvent-accessible surface area. **(E)** Number of intermolecular hydrogen bonds over time. **(F)** Per-residue RMSF. **(G)** Projected free-energy surfaces of the three replicate trajectories. Time is reported in ns; RMSD, interface RMSD, center-of-mass distance, Rg, and RMSF in nm; SASA in nm²; hydrogen bonds as counts; and projected relative free-energy values in kJ/mol. The three trajectories are independent computational replicates rather than biological replicates. Biological n, inferential hypothesis testing, multiplicity adjustment, and asterisk notation are therefore not applicable.

Interface RMSD showed greater replicate-dependent heterogeneity. Replicates 2 and 3 sampled broader interface RMSD values of approximately 0.70–1.00 nm after 40 ns, whereas replicate 1 remained within a narrower range of approximately 0.20–0.30 nm (**Figure 11A**), indicating that the three simulations sampled different interfacial conformational regimes. The center-of-mass separation between PPARG chain A and PTPN11 chain B remained within a comparatively narrow range in all three replicates (**Figure 11B**). R**_g_** and SASA showed only modest fluctuations across the trajectories, without sustained monotonic drift in either global descriptor (**Figures 11C, D**).

Intermolecular hydrogen bonds between PPARG and the PTPN11 construct were detected at multiple time points during the simulations (**Figure 11E**); this observation was treated only as a trajectory descriptor and not as evidence that hydrogen bonding maintained a physiological bound state. RMSF profiles were broadly similar between chains A and B, with larger fluctuations concentrated in loop and coil regions; several chain-B residues at the modeled interface nevertheless displayed higher RMSF values than their chain-A counterparts (**Figure 11F**). The projected free-energy surfaces displayed a dominant low-energy basin in each replicate, although basin breadth differed among trajectories (**Figure 11G**). Taken together, the simulations showed comparatively bounded global structural descriptors but heterogeneous interface-level motion; they do not establish stable physiological PPARG–PTPN11 binding.

### 3.11 Regulatory network construction, PPARG–ligand docking, and molecular dynamics analysis

TFs predicted to target the hub PMFRDEGs were retrieved from ChIPBase, and the corresponding regulatory pairs were imported into Cytoscape for construction of a TF-mRNA network. The network contained two hub genes together with 23 transcription factors (**Figure 12A**), with the corresponding annotations cataloged in **Supplementary Table S8**. For *Pparg*, CTD-derived chemical–gene associations were assembled into a 21-compound network (**Figure 12B**), and the underlying records are reported in **Supplementary Table S9**. These candidate compounds were then screened by protein–ligand docking against PPARG (**Figure 12C**), and the three compounds with the most favorable AutoDock Vina docking scores were selected for subsequent structural analysis.

**Figure 12:**
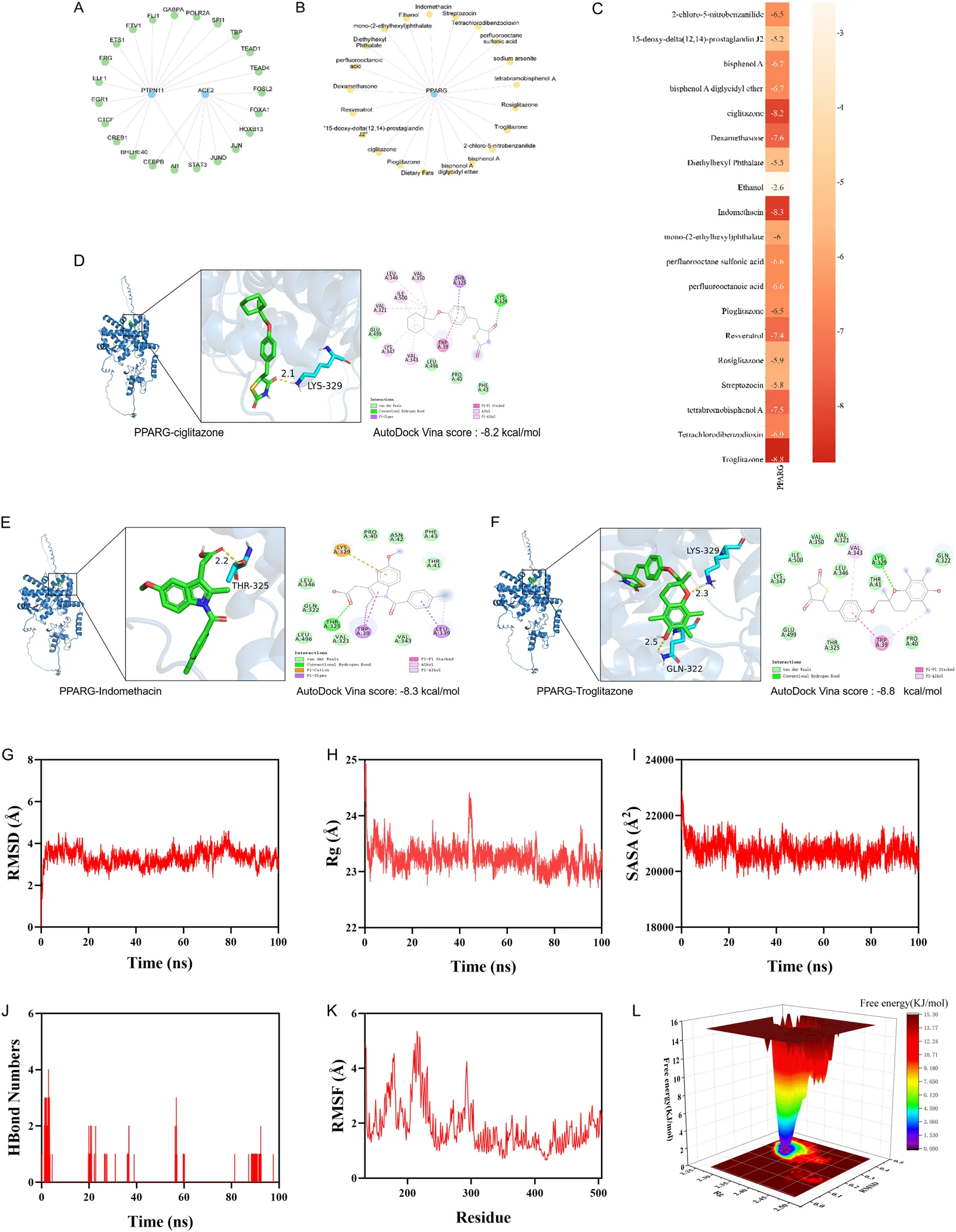
Regulatory networks, PPARG–ligand docking, and protein–ligand molecular dynamics analysis. **(A)** TF–mRNA regulatory network associated with the hub genes. **(B)** Drug–gene association network involving *Pparg*. **(C)** AutoDock Vina docking scores for PPARG-associated compounds, reported in kcal/mol. **(D–F)** Predicted docking poses of PPARG with ciglitazone, indomethacin, and troglitazone, respectively. **(G–L)** Molecular dynamics trajectory analyses of the modeled PPARG–troglitazone docking pose: **(G)** PPARG backbone RMSD, **(H)** radius of gyration, **(I)** solvent-accessible surface area, **(J)** intermolecular hydrogen-bond count, **(K)** protein RMSF, and **(L)** RMSD–Rg projected free-energy surface. Time is reported in ns; RMSD, Rg, and RMSF in Å; SASA in Å²; hydrogen bonds as counts; and projected relative free-energy values in kJ/mol. Biological n is not applicable because these panels represent network, docking, or molecular dynamics analyses. No conventional inferential statistical test or multiplicity adjustment was performed, and asterisk notation is not used. AutoDock Vina scores are computational docking scores rather than experimentally measured binding free energies.

In the highest-ranked ciglitazone pose, the ligand occupied the predefined PPARG docking region, and PLIP assigned a putative hydrogen-bond contact with Lys329. Additional hydrophobic, van der Waals, and π-related contacts were predicted around the modeled pocket (**Figure 12D**). In the indomethacin docking model, a hydrogen bond was predicted with Thr325, along with hydrophobic, van der Waals, π-σ, π-π stacking, and π-cation interactions among adjacent residues (**Figure 12E**). The troglitazone docking model likewise placed the ligand near Lys329, with a predicted hydrogen-bond contact and additional putative hydrophobic, van der Waals, and π-π stacking contacts within the predefined PPARG pocket (**Figure 12F**).

A separate protein–ligand molecular dynamics simulation was used to characterize the trajectory behavior of the predicted PPARG–troglitazone docking pose. After approximately 80 ns, the PPARG backbone RMSD fluctuated around 0.4 nm (**Figure 12G**). During the later part of the trajectory, PPARG R_g_ and SASA showed only modest variation, without sustained drift in these global protein-level descriptors (**Figures 12H, I**). Intermolecular hydrogen bonds were transiently detected during the trajectory, with no persistent hydrogen-bond network evident (**Figure 12J**). Most protein residues had RMSF values below 0.5 nm (**Figure 12K**). The RMSD–R_g_ projected free-energy surface contained a prominent low-energy basin near an RMSD of 0.32 nm and an R_g_ of 2.33 nm (**Figure 12L**). These trajectory descriptors characterize the sampled docked model but should not be interpreted as evidence of physiological binding or target engagement.

### 3.12 Establishment of an HFD/L-NAME-induced HFpEF murine model and validation of hub-gene expression

A murine model of HFpEF was induced by 15 weeks of HFD feeding together with L-NAME treatment (28). Body weight diverged progressively between groups, with significant effects of time (*P* < 0.0001), group (*P* = 0.00045), and the time-by-group interaction (*P* = 0.0023; **Figure 2A**). At the end of the intervention, systolic and diastolic blood pressures were higher in HFD/L-NAME-treated mice (both *P* < 0.0001), as was mean arterial pressure (*P* = 0.0022; **Figures 2B–D**). Running distance was markedly reduced (*P* < 0.0001), whereas the lung wet-to-dry weight ratio was increased (*P* = 0.0014; **Figures 2E, F**). Left ventricular ejection fraction (*P* = 0.9035) and fractional shortening (*P* = 0.7134) did not differ appreciably between groups (**Figures 2G–I**). The E/e′ ratio was higher after HFD/L-NAME exposure (*P* = 0.0496; **Figures 2J, K**). Histological quantification showed greater cardiomyocyte cross-sectional area (*P* = 0.0022) and myocardial fibrotic area (*P* = 0.0316; **Figures 2L–N**).

RT-qPCR showed lower myocardial transcript abundance of *Ptpn11* (*P* = 0.0276), *Pparg* (*P* = 0.0339), and *Ace2* (*P* = 0.0303) in HFpEF mice (**Figures 2O–Q**). At the protein level, Western blot densitometry showed reduced GAPDH-adjusted PTPN11 (*P* < 0.0001), PPARG (*P* < 0.0001), and ACE2 (*P* = 0.0004), corresponding to reductions of 89.1%, 80.4%, and 69.7%, respectively (**Figures 2R–U**). Immunohistochemical IOD measurements were likewise lower for PTPN11, PPARG, and ACE2 (all *P* < 0.0001), by 42.6%, 57.1%, and 67.5%, respectively (**Figures 2V–Y**). These effect sizes were interpreted within each assay: Western blotting quantified GAPDH-adjusted protein signals in whole-tissue lysates, whereas immunohistochemical IOD integrated DAB staining across sampled histological fields; the absolute values and fold changes from the two assays were therefore not treated as directly interchangeable.

### 3.13 Whole-blood *PTPN11* expression in patients with HFpEF

RT-qPCR analysis was conducted on whole-blood samples obtained from 19 patients with HFpEF and 18 control individuals. Compared with control participants, whole-blood *PTPN11* mRNA abundance was markedly lower in patients with HFpEF (*P* = 0.000051; **Figure 13A**). This direction was consistent with the exploratory HFpEF_2020 human left-ventricular dataset, in which *PTPN11* expression was also lower in HFpEF than in controls.

**Figure 13:**
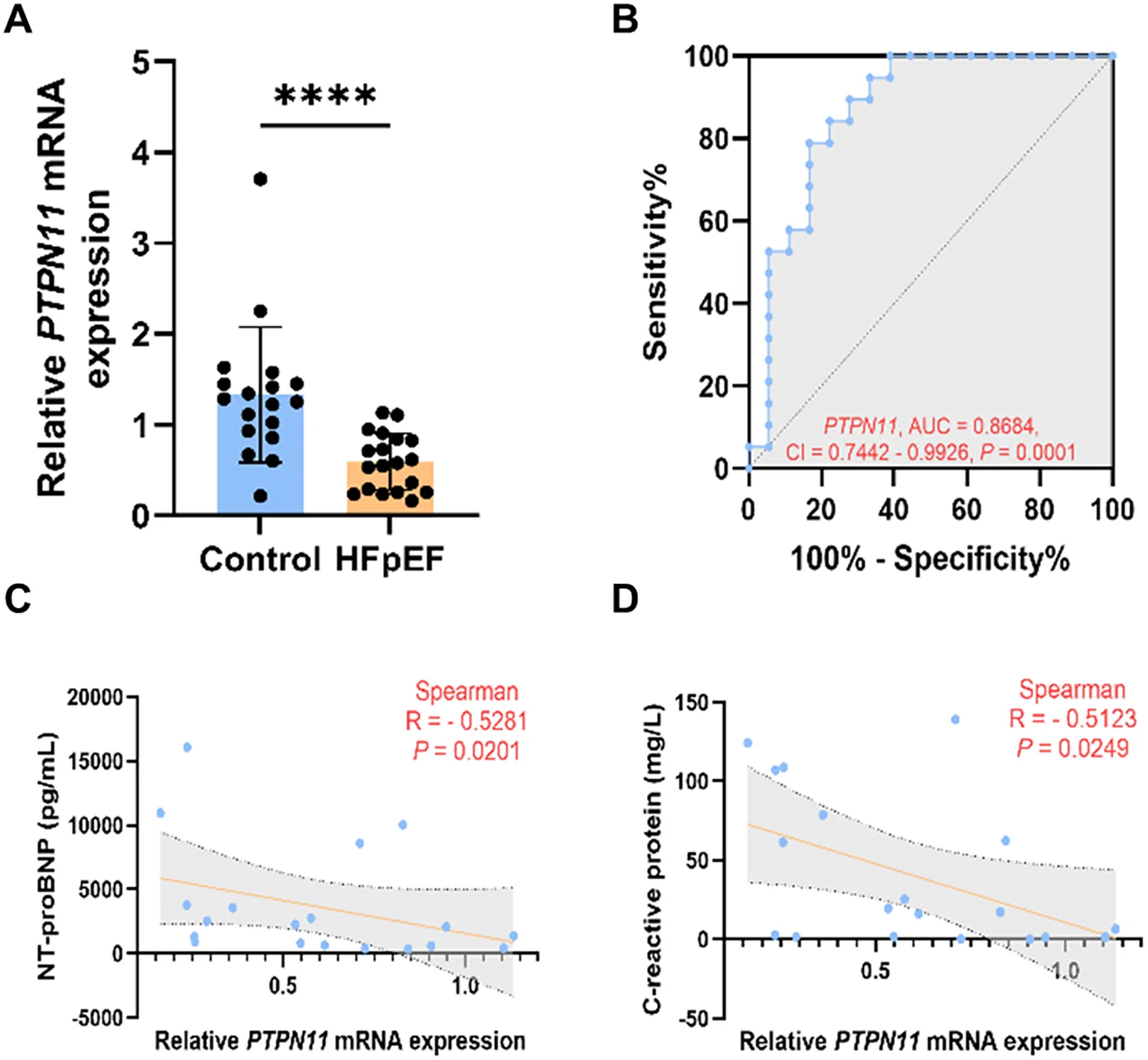
Exploratory whole-blood *PTPN11* analysis in patients with HFpEF. **(A)** Relative whole-blood *PTPN11* mRNA abundance (2^−ΔΔ^*^Ct^*; dimensionless) in controls (n = 18) and patients with HFpEF (n = 19), compared using a two-sided Mann–Whitney U test (*P* = 0.000051). **(B)** *Post hoc*, unadjusted ROC analysis using the same 37 participants; the AUC was 0.868 (95% CI, 0.744–0.993; *P* = 0.00013), with HFpEF treated as the positive clinical state and lower *PTPN11* abundance oriented toward HFpEF classification. **(C, D)** Two-sided Spearman’s rank correlations within the HFpEF group (n = 19) between whole-blood *PTPN11* mRNA abundance and NT-proBNP (pg/mL; C) or CRP (mg/L; D), respectively. Spearman’s *R* was −0.5281 (*P* = 0.0201) for NT-proBNP and −0.5123 (*P* = 0.0249) for CRP. Regression lines and 95% confidence bands, where displayed, are visual guides only; inferential conclusions are based on Spearman’s rank correlation rather than on the regression slope. No multiplicity adjustment was applied to panels A, C, or **(D)** In panel **(A)**, asterisks denote nominal two-sided *P* values according to the common convention: ns, *P* ≥ 0.05; ^∗^*P* < 0.05; ^∗∗^*P* < 0.01; ^∗∗∗^*P* < 0.001; ^∗∗∗∗^*P* < 0.0001. Asterisk notation is not used for panels **(B–D)**.

An exploratory, *post hoc* ROC analysis was performed to quantify separation of HFpEF from control status by whole-blood *PTPN11* mRNA in this small, unmatched cohort. The unadjusted AUC was 0.868 (95% CI, 0.744–0.993; *P* = 0.00013; **Figure 13B**). Within the HFpEF group, *PTPN11* mRNA abundance was inversely associated with NT-proBNP (Spearman’s *ρ* = −0.5281, 95% CI, −0.7976 to −0.0828; *P* = 0.0201) and CRP (*ρ* = −0.5123, 95% CI, −0.7896 to −0.0613; *P* = 0.0249; **Figures 13C, D**). These observations represent unadjusted, sample-level associations and do not establish that *PTPN11* is independently associated with HFpEF.

## 4 Discussion

In the present study, transcriptomic datasets were integrated to search for differentially expressed genes lying at the intersection of pyroptosis, myocardial fibrosis, and HFpEF. From this analytical pipeline, *Pparg*, *Ace2*, and *Ptpn11* were distilled into an exploratory three-gene candidate signature rather than an established clinical diagnostic model. Although the panel showed strong discrimination within the analyzed datasets, its clinical applicability requires confirmation in larger cohorts with prespecified validation procedures. Given the small discovery cohort, the attendant risk of overfitting, and heterogeneity in species, tissue source, and analytical context, the observed concordance is better regarded as preliminary replication than as evidence of transportability. Its biological plausibility was further supported by findings from the HFD/L-NAME mouse model and whole-blood samples obtained from patients with HFpEF. These results suggest a possible connection between inflammatory cell death and fibrotic remodeling in HFpEF and nominate *Pparg*, *Ace2*, and *Ptpn11* as exploratory diagnostic candidates; the stability of that nomination, however, remains to be tested in larger independent cohorts.

By integrating pyroptosis- and fibrosis-related gene sets, this study places two biologically plausible processes within a common HFpEF framework. The six-gene enrichment analysis highlighted SMAD binding, signaling-adaptor activity, autolysosome/amphisome terms, cellular senescence, efferocytosis, and renin-angiotensin signaling, which have been implicated in inflammatory injury and matrix remodeling (33). However, with only six PMFRDEGs, these annotations are sensitive to individual-gene contributions and do not establish mechanistic coupling between pyroptosis and fibrosis; perturbational and cell-resolved studies are required (8).

Among the three hub genes, *Pparg* appeared to have the most prominent role in the proposed molecular framework. *Pparg* encodes peroxisome proliferator-activated receptor gamma, a nuclear receptor involved in glucose and lipid metabolism, macrophage polarization, and transcriptional regulation of antifibrotic pathways (34). Its relevance to the metabolic and inflammatory features of HFpEF has also received increasing attention (35). In the present analyses, *Pparg* carried the largest point contribution in the nomogram and showed comparatively strong discrimination across the available datasets, although the small discovery sample leaves the magnitude and stability of that signal uncertain. These findings are consistent with the broad regulatory functions attributed to this gene. Reduced PPARG activity may promote metabolic stress, persistent inflammation, and profibrotic signaling. Over time, these changes could contribute to increased myocardial stiffness and impaired diastolic function (36). Previous experimental studies have reported that PPARG activation may attenuate cardiac fibrosis and slow HFpEF-related remodeling. PPARG-associated metabolic pathways have also been implicated in diabetic forms of HFpEF (37, 38). The drug-gene network, docking calculations, and molecular dynamics trajectories instead place PPARG among computationally prioritized candidates for ligand-focused follow-up; they do not, by themselves, establish druggability. These computational results, however, should be regarded as preliminary. Predicted docking poses and molecular-dynamics trajectory behavior do not demonstrate target engagement, biological efficacy, or clinical safety. This distinction is particularly important because some PPARG agonists have raised safety concerns in patients with heart failure (39).

Although *Ace2* and *Ptpn11* are involved in different biological pathways, they may represent complementary aspects of HFpEF within the exploratory three-gene panel. ACE2 is an established counter-regulatory component of the renin-angiotensin system and has been associated with anti-inflammatory and antifibrotic effects in the cardiovascular system (40). Its retention is consistent with the contribution of neurohormonal dysregulation to HFpEF. *Ptpn11* was a less expected candidate. It encodes PTPN11, a phosphatase that participates in growth-factor signaling, immune regulation, and tissue remodeling (41). In the original full-cohort feature-selection sequence, *Ptpn11* passed univariate screening and was subsequently retained by SVM-RFE and LASSO. That within-sample retention did not translate into robust resampling stability, however: *Ptpn11* was selected in only 38.4% of the nested outer training folds. It does not establish threshold robustness, because the analytical pipeline was not repeated under stricter effect-size cutoffs. *Ptpn11* also displayed a potentially informative discriminative signal and a biologically plausible connection to HFpEF-related pathways; its diagnostic contribution remains provisional, while the accompanying immune-cell associations are deconvolution-based and require separate cellular validation. Direct evidence tying PTPN11 specifically to HFpEF is still sparse. Even so, recent studies have placed this phosphatase within the regulation of inflammatory homeostasis and macrophage-driven tissue remodeling, offering a reasonable biological rationale for its inclusion in the model (42, 43). Viewed in this way, the three-gene signature may reflect three partially overlapping dimensions of HFpEF pathology: *Pparg*-linked metabolic and transcriptional regulation, *Ace2*-associated neurohormonal and antifibrotic control, and *Ptpn11*-related immune remodeling.

Immune-cell deconvolution was therefore treated as a hypothesis-generating layer rather than evidence of altered myocardial immune composition. The ImmuCC signature matrix is mouse-specific, but it is not myocardium-specific and has not been specifically validated in murine HFpEF myocardium. In the present dataset, none of the 23 nonzero inferred cell fractions differed between HFpEF and control after Benjamini–Hochberg correction; a statistically established shift in immune-cell composition cannot therefore be inferred from these data. Independent proteomic and experimental studies nevertheless place immune-inflammatory variation within the broader biology of HFpEF, including phenotype-associated proteomic patterns and T-cell inflammatory signaling (44, 45). The strong inverse correlation between M0 and M2 macrophage fractions may partly arise from the compositional constraint imposed by relative-fraction estimates rather than from biological polarization. The *Ptpn11*–mast-cell correlation is similarly tentative, particularly because mast-cell estimates derived from bulk myocardium are sensitive to reference-matrix fit and were not corroborated by direct cellular measurements. Flow cytometry, immunostaining, or single-cell or spatial profiling will be required before these patterns can be interpreted as changes in cell abundance or cell-specific biology.

Protein–protein interaction mapping, PPARG–PTPN11 docking, and three 100-ns molecular-dynamics replicates provide only a structural hypothesis. The truncated PTPN11 construct and heterogeneous interface motion preclude conclusions about native full-length binding, stability, functional coupling, or causality. The separate PPARG–ligand docking and PPARG–troglitazone simulations likewise do not demonstrate target engagement, druggability, or therapeutic efficacy. Because troglitazone was withdrawn for hepatotoxicity, these computations provide no therapeutic rationale for its use in HFpEF; biochemical binding and perturbational studies are required.

*In vivo* validation reproduced a cardiometabolic HFpEF phenotype after HFD/L-NAME treatment, with higher body weight and blood pressure, reduced exercise capacity, pulmonary congestion, elevated E/e′, cardiomyocyte enlargement, and myocardial fibrosis despite preserved systolic function (28, 46). Myocardial *Ptpn11*, *Pparg*, and *Ace2* transcripts and corresponding PTPN11, PPARG, and ACE2 proteins were reduced. This contrasted with the integrated murine discovery cohort, in which *Pparg* and *Ptpn11* were higher and *Ace2* lower, and with HFpEF_2020, where *PPARG* and *PTPN11* were lower while *ACE2* remained lower. Possible contributors include tissue and cellular composition, species, disease model or stage, sex distribution, and assay platform; transcript and protein assays also differ in normalization, dynamic range, and technical variability. These explanations remain tentative, and the inconsistent *Pparg*/*PPARG* and *Ptpn11*/*PTPN11* directions argue against a fixed cross-species expression score, whereas *Ace2*/*ACE2* showed a more coherent decrease. Whole-blood *PTPN11* interpretation is additionally limited by age, renal function, inflammatory status, medication exposure, and leukocyte composition; differential counts did not exclude confounding, and no multivariable adjustment was performed.

Accordingly, the three-gene panel should be viewed as hypothesis-generating rather than clinically established. The whole-blood *PTPN11* finding supports further study but does not establish diagnostic utility. Both the circulating signal and the three-gene signature require validation in larger, multicenter, independent prospective cohorts before clinical application. Any future added value should be demonstrated through incremental discrimination, calibration, and net clinical benefit beyond established clinical variables (47).

Several limitations remain. The discovery datasets differed in platform, sample handling, induction paradigm, treatment duration, group ratios, and sex composition; batch correction cannot remove this design heterogeneity. GSE194151 and sex-stratified sensitivity analyses did not stably reproduce the merged-cohort PMFRDEG pattern, whereas GSE180065 was too small for robust stand-alone replication. Model development was also vulnerable to overfitting: complete separation required Firth regression; bootstrap correction began after the three genes had been fixed; nested resampling reduced the mean out-of-fold AUC to 0.9286 and recovered the exact *Pparg*-*Ptpn11*-*Ace2* combination in only 13.2% of folds; and the six PMFRDEGs entering nested resampling had already been discovered from the full 45-sample cohort. The HFpEF_2020 analysis re-estimated coefficients and therefore provides panel-level replication rather than validation of a locked cross-species score. The permissive differential-expression screen was threshold-sensitive, so the six-gene pool and derived classifier remain hypothesis-generating rather than threshold-robust. GO/KEGG enrichment used only six genes and is sensitive to individual genes and annotation coverage. Immune deconvolution, regulatory-network analysis, docking, and molecular dynamics remain exploratory. The clinical cohort was small (19 HFpEF, 18 controls), unmatched, and unadjusted, leaving residual confounding. Finally, canonical pyroptosis readouts were not measured, candidate genes were not experimentally perturbed, and cell-type localization was unresolved; the data therefore cannot establish mechanistic coupling between pyroptosis and fibrosis or causal relationships among the candidate genes.

## 5 Conclusions

This exploratory study identified a compact three-gene molecular signature derived from pyroptosis- and myocardial fibrosis-related gene sets in HFpEF. The fixed three-gene Firth model retained high discrimination after bootstrap optimism correction, whereas repeated nested cross-validation yielded a lower out-of-fold estimate and exposed limited stability of the exact gene combination. Analysis of the HFpEF_2020 human left-ventricular dataset provided panel-level replication after model re-estimation; transportability of the signature nevertheless remains uncertain. In the clinical cohort, lower whole-blood *PTPN11* mRNA was associated with HFpEF status and with higher NT-proBNP and CRP levels, although these unadjusted observations require independent validation. Together, these findings provide a focused molecular framework in which fibrosis-related molecular signals coexist with exploratory immune-cell association patterns rather than demonstrated shifts in myocardial immune composition. Prospective cohorts and cell-resolved mechanistic studies will be required to determine the clinical and biological relevance of these candidate signals.

## Data Availability

The datasets presented in this study can be found in online repositories. The names of the repository/repositories and accession number(s) can be found in the article/**Supplementary Material**.

## Supplementary material

Supplementary Figure 1 Full, uncropped immunoblots corresponding to **Figure 2R**.

Supplementary Table 1 Original sample-level metadata exported from GEO for GSE194151.

Supplementary Table 2 Original sample-level metadata exported from GEO for GSE180065.

Supplementary Table 3 Final murine pyroptosis- and myocardial fibrosis-related gene intersection after human-to-mouse ortholog mapping (59 PMFRGs).

Supplementary Table 4 GO and KEGG enrichment results for PMFRDEGs.

Supplementary Table 5 Primer sequences used for amplification in mice.

Supplementary Table 6 Primer sequences used for amplification in humans.

Supplementary Table 7 Groupwise comparisons of 23 nonzero CIBERSORT-inferred immune-cell fractions using the ImmuCC reference matrix.

Supplementary Table 8 mRNA–TF regulatory network of model genes.

Supplementary Table 9 Drug prediction based on model genes.

Supplementary Data Sheet 1 Final murine pyroptosis-related gene set after homologene-based human-to-mouse ortholog mapping, removal of unmapped genes, and deduplication (n = 460).

Supplementary Data Sheet 2 Final murine myocardial fibrosis-related gene set after homologene-based human-to-mouse ortholog mapping, removal of unmapped genes, and deduplication (n = 611).

## Glossary

ACEI: angiotensin-converting enzyme inhibitor
ANOVA: analysis of variance
ARB: angiotensin II receptor blocker
ARNI: angiotensin receptor-neprilysin inhibitor
AUC: area under the curve
CIBERSORT: Cell-type Identification by Estimating Relative Subsets of RNA Transcripts
CRP: C-reactive protein
CTD: Comparative Toxicogenomics Database
DAB3: 3′-diaminobenzidine
DCA: decision curve analysis
DEGs: differentially expressed genes
EDTA: ethylenediaminetetraacetic acid
E/A: early diastolic mitral inflow velocity/atrial contraction mitral inflow velocity
E/e′: early mitral inflow velocity/early diastolic mitral annular velocity
eGFR: estimated glomerular filtration rate
FDR: false discovery rate
FPKM: fragments per kilobase of transcript per million mapped reads
GAFF2: General AMBER Force Field 2
GAPDH: glyceraldehyde-3-phosphate dehydrogenase
GEO: Gene Expression Omnibus
GO: Gene Ontology
GROMACSGR: Oningen MAchine for Chemical Simulations
H&E: hematoxylin and eosin
HFD: high-fat diet
HFpEF: heart failure with preserved ejection fraction
IOD: integrated optical density
KEGG: Kyoto Encyclopedia of Genes and Genomes
LASSO: least absolute shrinkage and selection operator
LINCS: LINear Constraint Solver
L-NAMEN: ^ω^-nitro-L-arginine methyl ester hydrochloride
log_2_FC: log_2_ fold change
LV: left ventricle
LVEF: left ventricular ejection fraction
LVFS: left ventricular fractional shortening
MFRGs: myocardial fibrosis-related genes
MM/GBSA: molecular mechanics/generalized Born surface area
mRNA: messenger RNA
mSVM-RFE: multiple support vector machine–recursive feature elimination
NPTconstant: number of particles pressure and temperature
NT-proBNP: N-terminal pro-B-type natriuretic peptide
NVTconstant: number of particles volume and temperature
NYHA: New York Heart Association
PCA: principal component analysis
PDB: Protein Data Bank
PDBQT: Protein Data Bank format containing partial atomic charges and AutoDock atom types
PLIP: Protein–Ligand Interaction Profiler
PME: particle mesh Ewald
PMID: PubMed identifier
PMFRDEGs: pyroptosis- and myocardial fibrosis-related differentially expressed genes
PMFRGs: pyroptosis- and myocardial fibrosis-related genes
PPI: protein–protein interaction
PRGs: pyroptosis-related genes
QC: quality control
QN: quantile normalization
RESP: restrained electrostatic potential
R_g,_: radius of gyration
RMSD: root mean square deviation
RMSE: root mean square error
RMSF: root mean square fluctuation
RNA-seq: RNA sequencing
ROC: receiver operating characteristic
R-SMAD: receptor-regulated SMAD
RT-qPCR: reverse transcription quantitative polymerase chain reaction
SASA: solvent-accessible surface area
SGLT2: sodium-glucose cotransporter 2
STRING: Search Tool for the Retrieval of Interacting Genes/Proteins
SUMO: small ubiquitin-like modifier
SVM-RFE: support vector machine–recursive feature elimination
TF: transcription factor
TIP3P: Transferable Intermolecular Potential with 3 Points
WBC: white blood cell count
