# Supplementary material for: Immune and inflammatory features associated with pyroptosis- and myocardial fibrosis-related genes in HFpEF: a machine learning study
Source: Front Immunol. 2026 Oct 2;17:1925067. doi: 10.3389/fimmu.2026.1925067 (PMC13672030; doi:10.3389/fimmu.2026.1925067)
Supplement: Supplementary file 1 [file Image1.pdf]

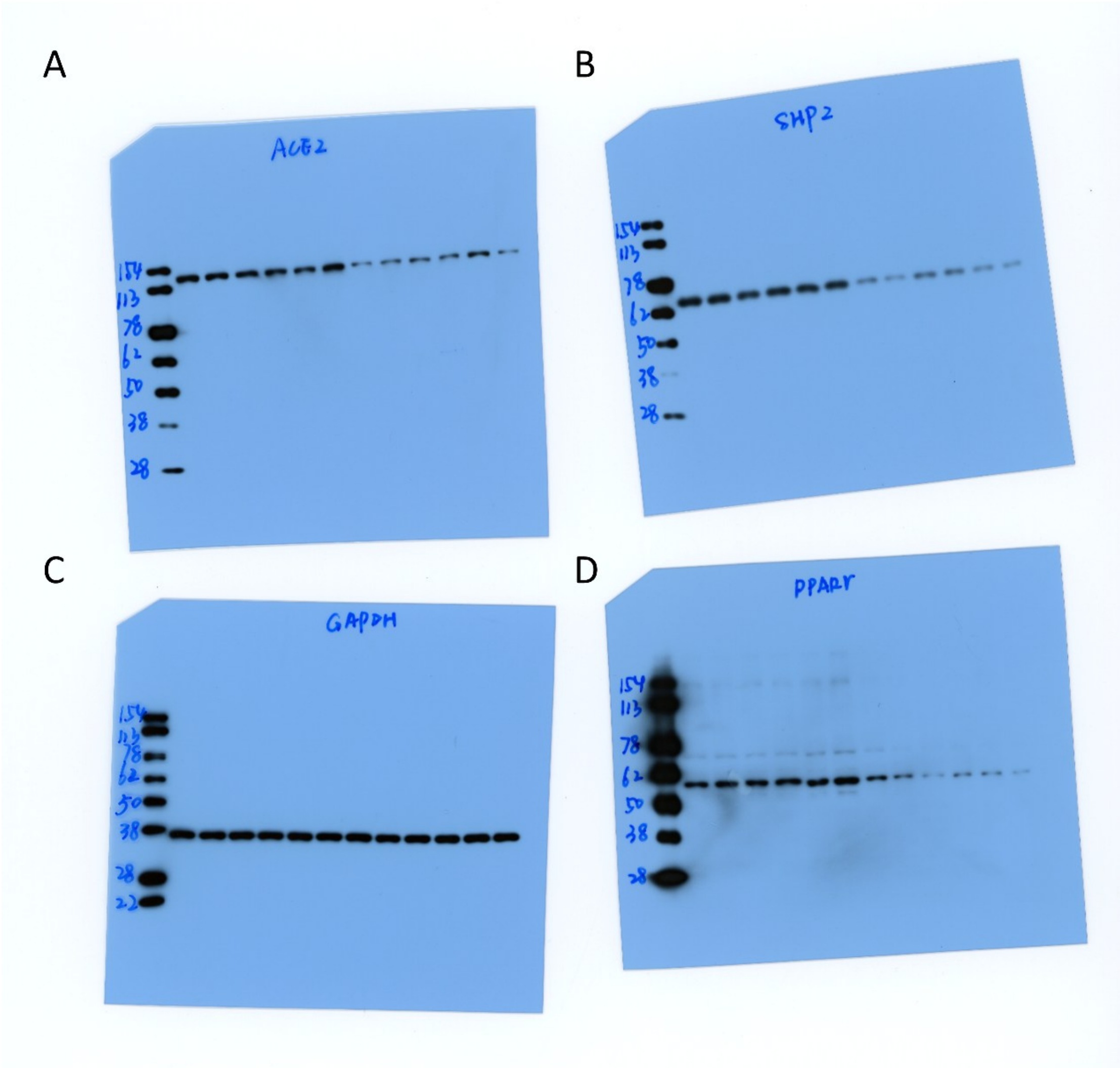

FIGURE S1

Full, uncropped immunoblots corresponding to Figure 2R. Western blot membranes showing protein expression in heart tissues from Control and HFpEF mice. Molecular weight markers (kDa) are indicated on the left. Each lane represents an individual biological replicate. The cropped images presented in Figure 2R were derived from these original blots. **(A)** Full, uncropped immunoblot for ACE2. **(B)** Full, uncropped immunoblot for SHP2 (PTPN11). **(C)** Full, uncropped immunoblot for GAPDH (loading control). **(D)** Full, uncropped immunoblot for PPARG.
