# Supplementary material for: Immune and inflammatory features associated with pyroptosis- and myocardial fibrosis-related genes in HFpEF: a machine learning study
Source: Front Immunol. 2026 Oct 2;17:1925067. doi: 10.3389/fimmu.2026.1925067 (PMC13672030; doi:10.3389/fimmu.2026.1925067)
Supplement: Supplementary file 2 [file Table4.docx]

Supplementary Table 4. GO and KEGG enrichment results for PMFRGs.

| ONTOLOGY | ID | Description | GeneRatio | BgRatio | pvalue | p.adjust | qvalue |
| --- | --- | --- | --- | --- | --- | --- | --- |
| BP | GO:0050886 | endocrine process | 3/6 | 113/28891 | 1.16E-06 | 9.00E-04 | 1.57E-04 |
| BP | GO:0001659 | temperature homeostasis | 3/6 | 195/28891 | 5.97E-06 | 2.32E-03 | 4.05E-04 |
| BP | GO:0034764 | positive regulation of transmembrane transport | 3/6 | 282/28891 | 1.80E-05 | 2.52E-03 | 4.39E-04 |
| BP | GO:2000272 | negative regulation of signaling receptor activity | 2/6 | 34/28891 | 2.01E-05 | 2.52E-03 | 4.39E-04 |
| BP | GO:0030072 | peptide hormone secretion | 3/6 | 321/28891 | 2.65E-05 | 2.52E-03 | 4.39E-04 |
| CC | GO:0090575 | RNA polymerase II transcription regulator complex | 2/6 | 249/28573 | 1.11E-03 | 1.82E-02 | 4.95E-03 |
| CC | GO:0071141 | SMAD protein complex | 1/6 | 10/28573 | 2.10E-03 | 1.82E-02 | 4.95E-03 |
| CC | GO:0044753 | amphisome | 1/6 | 12/28573 | 2.52E-03 | 1.82E-02 | 4.95E-03 |
| CC | GO:0106068 | SUMO ligase complex | 1/6 | 13/28573 | 2.73E-03 | 1.82E-02 | 4.95E-03 |
| CC | GO:0044754 | autolysosome | 1/6 | 14/28573 | 2.94E-03 | 1.82E-02 | 4.95E-03 |
| MF | GO:0070412 | R-SMAD binding | 2/6 | 26/28407 | 1.21E-05 | 1.07E-03 | 1.40E-04 |
| MF | GO:0046332 | SMAD binding | 2/6 | 85/28407 | 1.32E-04 | 4.19E-03 | 5.45E-04 |
| MF | GO:0035591 | signaling adaptor activity | 2/6 | 88/28407 | 1.41E-04 | 4.19E-03 | 5.45E-04 |
| MF | GO:0019902 | phosphatase binding | 2/6 | 243/28407 | 1.07E-03 | 1.70E-02 | 2.21E-03 |
| MF | GO:0031625 | ubiquitin protein ligase binding | 2/6 | 338/28407 | 2.05E-03 | 1.70E-02 | 2.21E-03 |
| KEGG | mmu04380 | Osteoclast differentiation - Mus musculus (house mouse) | 2/6 | 136/9577 | 2.89E-03 | 8.96E-02 | 7.58E-02 |
| KEGG | mmu04148 | Efferocytosis - Mus musculus (house mouse) | 2/6 | 163/9577 | 4.13E-03 | 8.96E-02 | 7.58E-02 |
| KEGG | mmu04218 | Cellular senescence - Mus musculus (house mouse) | 2/6 | 183/9577 | 5.18E-03 | 8.96E-02 | 7.58E-02 |
| KEGG | mmu05205 | Proteoglycans in cancer - Mus musculus (house mouse) | 2/6 | 204/9577 | 6.40E-03 | 8.96E-02 | 7.58E-02 |
| KEGG | mmu04614 | Renin-angiotensin system - Mus musculus (house mouse) | 1/6 | 37/9577 | 2.30E-02 | 1.41E-01 | 1.19E-01 |

GO，Gene Ontology；BP，Biological Process；CC，Cellular Component；MF，Molecular Function；KEGG，Kyoto Encyclopedia of Genes and Genomes；PMFRDEGs，Pyroptosis and Myocardial Fibrosis-Related Differentially Expressed Genes。
